# Understanding and Reducing Appearance Anxiety for Social Media Users: A Study Using Ecological Momentary Assessment and Ecological Momentary Intervention

**DOI:** 10.1155/da/7212945

**Published:** 2026-06-15

**Authors:** Rong Chen, Jin Tong Bai, Yue Liu

**Affiliations:** ^1^ Institute of Brain and Psychological Sciences, Sichuan Normal University, Chengdu, Sichuan, China, sicnu.edu.cn; ^2^ Chengdu College of Arts and Sciences, Chengdu, Sichuan, China

**Keywords:** appearance anxiety, dynamic structural equation modeling, ecological momentary assessment, ecological momentary intervention, social media use

## Abstract

Appearance anxiety for social media users (AASMU) is emotional distress linked to social media use (SMU), negatively affecting mental and physical health. However, little is known about the dynamic relationship between AASMU and SMU, and effective interventions. Study 1 used Ecological Momentary Assessment (EMA) with 206 participants completing daily surveys for 20 days. Dynamic structural equation modeling (DSEM) revealed that AASMU tended to persist from 1 day to the next (*β* = 1.45) and that higher levels of AASMU and SMU often occurred together on the same day (*β* = 2.57). A higher level of self‐compassion (SC) or a healthier lifestyle moderated this relationship, providing resilience against SMU’s negative effects (*β* = −0.40 or −0.41). Study 2 utilized an ecological momentary intervention (EMI) with 200 participants, where the intervention group read SC statements daily. Results showed a significant reduction in both the mean level (*β* = −0.05) and intra‐individual variability of AASMU in the intervention group (*β* = −0.11), suggesting SC‐based EMI is an effective approach to reducing AASMU.

## 1. Introduction

With the widespread adoption of the Internet, social media has become a dominant force in the digital landscape [1]. Currently, the rise of mobile devices and technological advances has shifted social media to mobile platforms, giving rise to highly visual social media, which emphasizes user‐generated visual content, such as images and videos. Research suggested that frequent use of highly visual social media (e.g., Instagram) is linked to heightened appearance anxiety [2].

Two theoretical frameworks help explain appearance anxiety for social media users (AASMU). Objectification theory suggests that media‐induced self‐surveillance can lead to anxiety [3], while sociocultural theory highlights the role of social comparison and internalization of idealized beauty standards [2]. AASMU is therefore defined as a transient emotional experience of state anxiety during social media use (SMU), driven by concerns about body image and fear of negative evaluations due to exposure to appearance‐related content.

Based on its definition, AASMU is likely to be associated with short‐term social media usage patterns [4]. On the one hand, problematic SMU has been identified as a factor contributing to negative body image and appearance anxiety [5]. On the other hand, appearance anxiety can lead to increased SMU as individuals dissatisfied with their body image may use social media to strategically manage self‐presentation through pictures, selfies, videos, or other visual content [6]. Together, these findings support a bidirectional association between SMU and appearance anxiety. In addition, understanding this relationship also requires cultural consideration. In collectivist cultures like China, norms emphasizing conformity and social harmony may intensify the internalization of appearance ideals through social comparison [7]. Platforms such as Rednote (xiaohongshu) often present beauty as a form of social responsibility, reinforcing the perception that appearance management is expected for group acceptance. Compared to Western cultures that prioritize individuality, Chinese users may align their self‐presentation with idealized beauty standards promoted through homogenized and commercially mediated imagery [8]. Consistent with the objectification theory, such sociocultural pressures foster self‐surveillance and internalized evaluation, heightening appearance‐related anxiety.

Most previous studies examining AASMU and SMU have relied on cross‐sectional designs, which are limited in capturing the dynamic and reciprocal nature of their relationship. In reality, both social media engagement and the resulting AASMU are not stable traits but fluctuating states that vary across days or even within a single day, influenced by exposure to specific social media content, interactive features, or the user’s immediate affective state [9]. Traditional designs therefore lack the temporal resolution needed to examine these short‐term emotional processes and their causal interplay [10]. In addition, prior research has shown that socio‐environmental comparison (SEC), healthy lifestyle (HL), and self‐compassion (SC) are associated with emotional well‐being [11–14]. However, a crucial gap remains in understanding how these characteristics moderate the momentary relationship between SMU and fluctuations in appearance anxiety.

To precisely address these methodological limitations, the current study adopted an ecological momentary assessment (EMA) approach. This method directly provides more ecological and fine‐grained assessments of individuals’ daily experiences, overcoming recall bias and capturing the short‐term dynamics in SMU and AASMU that traditional methods cannot detect [15]. For example, recent EMA evidence suggests that momentary emotion dysregulation and fluctuations in self‐concept clarity are key processes underlying the affective consequences of social media engagement [16]. Moreover, EMA data can offer a strong empirical foundation for developing personalized, just‐in‐time adaptive interventions within the ecological momentary intervention (EMI) framework [17, 18].

AASMU have adverse effects on individuals’ mental and physical health. It is associated with lower self‐esteem, heightened body dissatisfaction, and elevated symptoms of anxiety, depression, and eating disorders [19]. AASMU also correlates with negative interpersonal traits such as suspiciousness and conformity [20] and may increase the likelihood of somatoform symptoms and psychiatric conditions like anorexia nervosa [21]. These challenges highlight the urgent need to explore effective interventions for AASMU.

**Figure 1:**
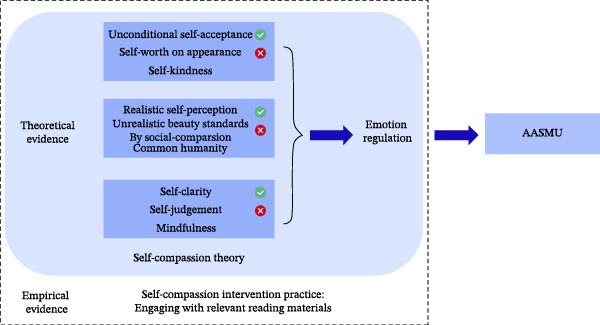
Theoretical and empirical evidence for self‐compassion‐based Intervention.

In response to this critical need, SC has emerged as a promising strategy for managing appearance anxiety, serving as a valuable theoretical basis for targeted interventions aimed at reducing AASMU [22]. Theoretically, SC mitigates maladaptive self‐evaluative processes, fostering a stable, accepting self‐concept and buffering social comparison and self‐criticism through enhanced emotion regulation [23] and reduced self‐judgment [24]. Its three core components systematically challenge appearance anxiety. Self‐kindness fosters unconditional self‐acceptance, decoupling self‐worth from physical perfection and external validation [24–26]. Common humanity counters isolating, self‐critical beliefs by contextualizing imperfections within universal human experience, de‐idealizing unrealistic beauty standards, and promoting realistic self‐perception [24, 26, 27]. Mindfulness [28] cultivates non‐judgmental awareness of appearance‐related thoughts [23], enabling cognitive defusion [29]. This reframe of anxious beliefs as transient mental events disrupts automatic reactions, thereby weakening their conviction. Empirically, a substantial body of literature supports the efficacy of SC interventions in ameliorating body dissatisfaction and appearance anxiety. Brief interventions, including SC‐focused reading materials and online psychoeducation modules, have consistently reduced these concerns [30, 31]. Further, text message‐based interventions have similarly improved body image [32]. Meta‐analyses corroborate this efficacy, demonstrating significant reductions in body dissatisfaction, body shame, and eating disorder symptoms [33, 34], underscoring their robust potential as accessible and scalable strategies. The theoretical framework of how SC alleviates AASMU is illustrated in Figure 1.

However, existing interventions remain limited in several ways. First, most SC interventions primarily address general body dissatisfaction rather than AASMU. Second, they are often implemented as single‐session, lab‐based treatments, which are resource‐intensive and lack ecological validity for daily life. Third, prior studies have focused mainly on mean‐level changes in appearance anxiety while overlooking dynamic indicators such as autoregressive patterns (emotional inertia) and intra‐individual variability (IIV), which are theoretically and clinically meaningful markers of emotional regulation [35–37].

To address these limitations, the present study implemented an EMI grounded in SC theory to reduce AASMU. By delivering brief daily interventions in participants’ natural environments, this approach allows day‐to‐day engagement with self‐evaluative and emotional vulnerabilities that arise during SMU. Such a design enhances ecological validity while maintaining a low participant burden. Prior research has demonstrated the effectiveness of EMIs in reducing depression, anxiety, and stress [38, 39], yet studies specifically applying this approach to AASMU remain scarce.

Furthermore, the present study is focused exclusively on women for both theoretical and empirical reasons. First, epidemiological evidence shows that women are ~2.3 times more likely than men to experience clinical levels of body dysmorphic symptoms [40]. Second, gendered sociocultural pressures disproportionately target women’s appearance through objectification and media portrayals, making them particularly vulnerable to appearance anxiety [41]. Third, neurobiological research suggests sex differences in visual processing of self‐related images, with women showing greater sensitivity to graphic content, which increases their susceptibility to appearance‐related cues on social media [42].

To evaluate both short‐term dynamics and intervention effects, we employed dynamic structural equation modeling (DSEM). This approach integrates time‐series modeling, multilevel SEM, and latent variable techniques to capture both within‐ and between‐person processes. Compared to standard generalized linear mixed modeling (GLMM), DSEM can accommodate intensive longitudinal data (ILD), handle unequal time intervals and missingness, and model autoregressive, cross‐lagged, and person‐specific effects [43]. These advantages make DSEM especially well‐suited for analyzing EMA and EMI data [44].

Building on this methodological framework, the primary purpose of this study is twofold. Study 1 aims to explore the dynamic relationship between AASMU and SMU by using DSEM based on data collected through an EMA design. Study 2 examines the effectiveness of reading self‐compassionate materials in reducing AASMU based on an EMI design. The intervention effects will be assessed not only in terms of changes in mean levels but also in carryover effects and IIV, again utilizing DSEM. We hope this study will shed lights on the dynamic process underlying AASMU and identify effective EMI‐based intervention strategies for mitigating AASMU.

## 2. Methods

### 2.1. Study Procedure

Study 1 employed EMA to investigate the underlying mechanisms and dynamic process between AASMU and SMU. The study began by collecting baseline data on time‐invariant variables for participants (including HL, SC, and social comparison) 3 days prior to the start of the EMA phase. Participants were then asked to complete EMA questionnaires for 20 consecutive days at 9 pm via *WenJuanXing* (an online platform). Nonresponses or late responses (submitted after 12 am) were coded as missing. Participants also rated their conscientiousness in completing the EMA measures on a scale of 1–100 each day.

**Figure 2:**
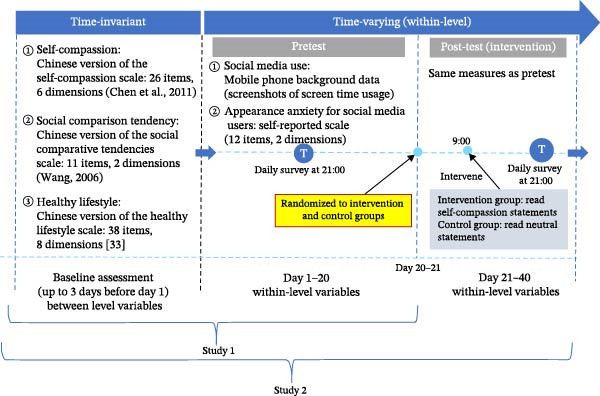
Study flowchart.

Study 2 examined the efficacy and feasibility of reading SC‐related statements as an intervention to reduce AASMU using an EMI design. Over 40 days, the first 20 served as a pretest (identical to Study 1), followed by a 20‐day intervention period. During the intervention period, time‐varying variables were continuously measured using the same EMA questionnaires as in Study 1. After the pretest period, participants were randomly assigned in equal sample sized to the intervention or control group using **RRApp** [45], a validated online randomization tool that ensures independent and concealed allocation. Then, the intervention group read self‐compassionate statements, while the control group read neutral statements. Subjects in both groups reviewed the material and submitted their readings via WeChat voice chat. To ensure engagement with the intervention, all participants rated their seriousness level on a scale of 1–100 after submitting their readings. Participants who scored below 60 were issued a warning from the research assistant to “answer carefully” in the subsequent days. The entire experimental procedure is depicted in Figure 2.

### 2.2. Participants Recruitment and Eligibility Criteria

Drawing on recommendations and sample size practices from ~50 previous studies that employed DSEM for analyzing EMA data (e.g., [46]), as well as the results of a Monte Carlo simulation–based power analysis conducted for our EMA and EMI study (see Tables S1 and S2 in the Supporting Information), we determined that a sample size of at least 200 participants with 20 daily assessments before and 20 daily assessments during the EMI period would provide adequate statistical power and estimation precision.

Eligibility criteria for participation are as follows: (1) female, aged 18–35, in good physical and mental health, with no history of neurological or mental disorders; (2) possession of a mobile phone with a system capable of tracking daily screen time; and (3) regular use of social media on mobile devices. During recruitment, participants were informed of the importance of adhering responsibly to the experimental protocol and of ensuring that they could meet the study’s daily task requirements. Consistent with previous studies (e.g., [47]), Exclusion criteria include: (1) extremely high or low levels of trait appearance anxiety, defined as deviating more than three standard deviations (SDs) from the sample mean of the Social Appearance Anxiety Scale (SAAS); (2) abnormally high or low body mass index (BMI) scores, defined as deviating more than 3SDs from the sample mean.

This study was approved by the Ethical Committee of Sichuan Normal University (Approval Number: 2022‐LS‐015). All procedures were conducted in accordance with institutional ethical guidelines and the Declaration of Helsinki. Prior to participation, individuals were presented with detailed electronic information regarding the study’s purpose, procedures, data protection policies, and their rights as participants.

**Figure 3:**
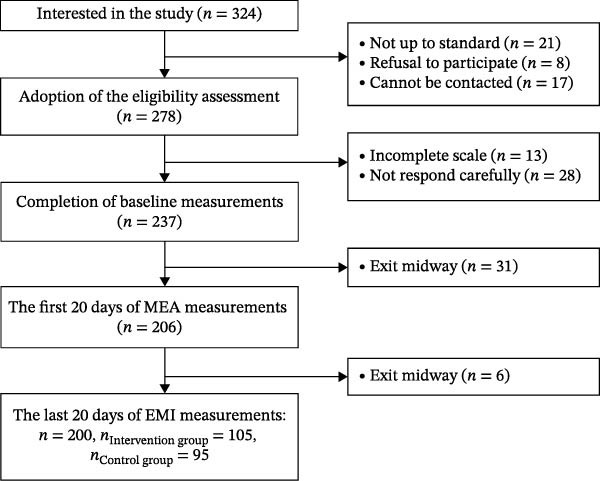
Flowchart of subjects’ participation.

Of the 324 individuals who completed the eligibility questionnaire, 21 did not meet the inclusion criteria, 8 declined to participate, and 17 were unavailable (see Figure 3). From the remaining 278 potential participants, 265 completed the pre‐test assessment (measuring time‐invariant variables). None was excluded as received warnings of “answer carefully.” However, 28 participants were excluded for being classified as “careless respondents,” defined as those with total response time less than 25% of the mean or who answered all three polygraph questions incorrectly (“I have never used a smartphone,” “please choose the third option for this question,” and “please skip this question”). In total, 237 participants met the experimental requirements and completed the pretest questionnaire honestly.

### 2.3. Measures

#### 2.3.1. Survey Measures

The survey included several time‐invariant variables assessed prior to the EMA study, including social comparison, HL, and SC.

1. Social comparison (SC): The Chinese version of the Social Comparison Tendency Scale (SCTS) was used to assess individuals’ tendency and ability to engage in social comparison. Specifically, the 11‐item version developed by Wang et al. [48] was employed, which includes two dimensions: competence (e.g., “I’m not the kind of person who often compares myself to others”) and viewpoints (e.g., “I always wonder what others would do in similar situations”). Items were rated on a 5‐point scale (1 = Not at all to 5 = Absolutely) and summed to produce an index of social comparison.
2. HL: The Health Lifestyle Scale (HLS) developed by Wang [49] included 8 dimensions and a total of 38 items. These dimensions covered sports and exercise behaviors (e.g., “I exercise vigorously half an hour after eating”), regular living patterns (e.g., “I live and work regularly”), dietary and nutritional habits (e.g., “I drink at least about 800 ml of water a day”), health risk behaviors (e.g., “I’m a smoker”), health responsibility (e.g., “I brush my teeth morning and night”), interpersonal support (e.g., “I’m helpful”), stress management (e.g., “I’ll watch my mood swings”), and appreciation of life (e.g., “I’m optimistic about my life.”). Items were rated on a 5‐point scale (1 = Not at all to 5 = Absolutely) and summed to produce an index of a HL.
3. SC: The Chinese adaptation of the Self‐Compassion Scale (SEC) by Hou [50] comprised 30 items across six dimensions: self‐kindness (e.g., “I try to be loving towards myself when I’m feeling emotional pain”), common humanity (e.g., “When things are going badly for me, I see the difficulties as part of life that everyone goes through”), mindfulness (e.g., “When something upsets me, I try to keep my emotions in balance”), self‐judgment (e.g., “I’m disapproving and judgmental about my own flaws and inadequacies”), isolation (e.g., “When I’m feeling down, I tend to feel like most other people are probably happier than I am”), and over‐identification (e.g., “When I’m feeling down I tend to obsess and fixate on everything that’s wrong”). Items were rated on a 5‐point scale (1 = Not at all to 5 = Absolutely) and summed to produce mean index of SC.

The reliability and validity indices for SC, HL, and SEC are provided in the Supporting Information. Based on previous studies, HL and SEC have been shown to influence emotional states such as anxiety [11–13]. Thus, SEC or HL was included in the model to predict the dynamic parameters at the within‐person level and the means of AASMU. Moreover, since previous studies have shown that SC also affects AASMU [14], SC, HL, and SEC were incorporated to predict the means of AASMU.

#### 2.3.2. EMA Measures

The EMA measures included daily assessments of time‐varying variables, including the AASMU and SMU.

1. AASMU: AASMU was measured using a self‐developed 17‐item scale comprising two dimensions: anxiety experience (e.g., “I’d be nervous about taking a photo that would be posted on social media”) and body monitoring (e.g., “I’ll check my social media posts about my appearance”). To reduce participant burden in EMA, we selected the 12 items with the highest factor loadings (six per dimension) based on CFA results. All items were rated on a 100‐point scale (1 = Not at all to 100 = Absolutely), which enhances sensitivity to within‐person fluctuations in daily anxiety levels and is consistent with prior EMA research practices (e.g., [51]). This scale demonstrated good internal consistency (Cronbach’s α = 0.85) and retest reliability (*r* = 0.75) and an acceptable model fit for a second‐order factor model comprising two first‐order factors (χ^2^(*N* = 608) = 1909.55, CFI = 0.93, TLI = 0.91, RMSEA = 0.06). The between‐person and within‐person reliabilities for AASMU are 0.94 and 0.81, respectively [52]. Full development and validation procedures for the original 17‐item AASMU scale are detailed in the Supporting Information.
2. Time of SMU: To objectively measure the participants’ daily usage of each social media platform, we collected screenshots of the daily screen usage time recorded by participants’ mobile phone systems. These screenshots provided the total time spent on each social media platform. An example of a screen usage time screenshot can be found in Figure S1 of the Supporting Information.

To justify the use of composite scores in subsequent DSEM analyses, CFAs were conducted for all multidimensional scales (AASMU, SC, HL, and SEC) using second‐order factor models. Each model demonstrated acceptable fit indices (see Supporting Information), supporting the structural validity of the overall scale scores used in the main analyses. Moreover, all measures, except for the self‐developed AASMU scale, were adapted from previously published Chinese versions that had undergone translation, back‐translation, and psychometric validation in prior research [48–50]. Therefore, no additional translation procedures were required in this study.

### 2.4. Intervention Materials

A total of 10 self‐compassionate and 10 neutral statements (each 100–150 words) were used during the intervention. The development procedure and full text of the reading materials used in the EMI are provided in the Supporting Information. An example of a SC statement is: “When feeling emotionally low, I approach myself with curiosity and openness, focusing on my present feelings.”

## 3. Study 1: The EMA Study

### 3.1. Statistical Analysis

**Figure 4:**
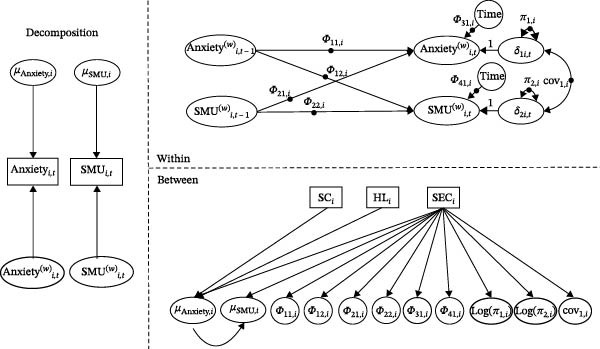
Analytical model of the dynamic relationship between the different levels of each variable in Model 1. Latent variables are represented by circles, while observed variables are represented by squares. (*w*) denotes the estimate at the time‐point level (within‐person level), with the subscript *i* denotes the subject, *t* denotes the time point and μ denotes the mean. AASMU and SMU represent the variables appearance anxiety for social media users and social media use respectively, while SC, HL, and SEC represent: social comparison tendency, healthy lifestyle, and level of self‐compassion, respectively. Solid black dots on each path indicate that the corresponding coefficients are random.

To examine the effects of the two protective factors: SC and HL separately, we estimated two DSEM models. Each model decomposed the data into within‐person (day‐level) and between‐person (individual‐level) components, as illustrated in the left panels of Figure 4 and Figure S2. Here, *μ*_AASMU,*i*_ and *μ*_SMU,*i*_ represent the within‐person means for AASMU and SMU, respectively. The temporal deviations from these means, ${\text{AASMU}}_{i.t}^{\left(w\right)}$ and ${\text{SMU}}_{i.t}^{\left(w\right)}$, represent the states of person *i* at time point *t*. The top right panel of the figure shows the cross‐lagged relationships between AASMU and SMU at the within‐person level. The bottom right panel illustrates the effects of between‐person (time‐invariant) variables (i.e., SC, HL, and SEC) on within‐person level coefficients (i.e., autoregressive coefficients, cross‐lagged coefficients, and residual variance and covariance). To improve interpretability and reduce multicollinearity, all between‐person predictors were grand‐mean centered by subtracting the overall sample mean from each individual’s raw score (i.e., grand‐mean centering).

Latent variables are represented by circles, while observed variables are represented by squares. *w* denotes the estimate at the time‐point level (within‐person level), with the subscript *i* denotes the subject, *t* denotes the time point, and *μ* denotes the mean. AASMU and SMU represent the variables AASMU and SMU, respectively, while SC, HL, and SEC represent social comparison tendency, HL, and level of SC, respectively. Solid black dots on each path indicate that the corresponding coefficients are random.

#### 3.1.1. Within‐Person Level Model

The within‐person level model captures the dynamic change and influence process of within‐person level variables ${\text{AASMU}}_{i.t}^{\left(w\right)}$and ${\text{SMU}}_{i.t}^{\left(w\right)}$over time. The equations of within‐person model in Model 1 and Model 2 were the same.

(1) AASMUi.tw=μAASMU.i+ϕ11.iAASMUi.t−1w+ϕ12.iSMUi.t−1w+ϕ13.iTime+δ1i.t,

(2) SMUi.tw=μSUM,i+ϕ22,iSUMi,t−1w+ϕ21,iAASMUi.t−1w+ϕ23,iTime+δ2i,t,

where *ϕ*_11.*i*_ and *ϕ*_22,*i*_ represent the autoregressive effects of AASMU and SMU, respectively. *ϕ*_12.*i*_ represents the cross‐lagged regression effect from SMU to AASMU at the next time point, while *ϕ*_21,*i*_ represents the cross‐lagged regression effect from AASMU to SMU. *ϕ*_13.*i*_ and *ϕ*_23,*i*_ capture time effects on the current state of AASMU and SMU, respectively, which are used for detrending [53]. *δ*_1*i*.*t*_ and *δ*_2*i*,*t*_ are the residual terms for AASMU and SMU, often referred to as innovations. These innovations are assumed to follow a multivariate normal distribution:

(3) δ1i,tδ2i,t∼MVN0, ∑i,

where *Σ*_**i**_ is the between‐person level covariance matrix, assumed to vary across individuals (random effect). Thus, the log of the variance of the innovations (i.e., log(*π*_1.*i*_) and log(*π*_2.*i*_), where *π*_1.*i*_ and *π*_2.*i*_ are the random variance of the innovations) and the covariance of the innovations (i.e., cov_1,*i*_) are also assumed to be random.

#### 3.1.2. Between‐Person Level Model

The between‐person‐level model reflects the effects of between‐person‐level variables on within‐person level variability. Therefore, the between‐person level model in Model 1 is expressed as:

(4) μAASMU,i=γ01+γ010SCi+γ011HLi+γ012SECi+u01i,

(5) μSUM,i=γ02+γ021SECi+u02i,

(6) ϕ11,i=γ10+γ11SECi+u11i,

(7) ϕ12,i=γ20+γ21SECi+u12i,

(8) ϕ21,i+γ30+γ31SECi+u21i,

(9) ϕ22,i=γ40+γ41SECi+u22i,

(10) ϕ13,i=γ50+γ51SECi+u13i,

(11) ϕ23,i=γ60+γ61SECi+u23i,

(12) logπ1.i=γ70+γ71SECi+uπ1.i,

(13) logπ2.i=γ80+γ81SECi+uπ2.i,

(14) cov1.i=γ90+γ91SECi+ucov.i,

where *γ*_010_, *γ*_011_, and *γ*_012_ represent the fixed effects of SC, HL, and SEC on AASMU, respectively. *γ*_021_ represents the fixed effect of HL on individuals’ SMU, *γ*_11_ and *γ*_41_ represents the fixed effect of SEC on the autoregressive effect of AASMU and SMU, *γ*_21_ and *γ*_31_ represent the fixed moderated effects of SEC on the cross‐lagged relationship between AASMU and SMU, *γ*_51_ and *γ*_61_ represent the fixed moderated effects of SEC on the time effects of AASMU and SMU, respectively, *γ*_71_ and *γ*_81_ denote the fixed effects of SEC on the random variance of the residuals of AASMU and SMU, respectively, and *γ*_91_ denotes the fixed effect of SEC on the random covariance of the residuals of AASMU and SMU. Note that in Model 2, SEC in Equations (5)–(14) were replaced with HL, which investigated the moderating effects of HL.

#### 3.1.3. Analysis

Data cleaning was conducted before model fitting. First, participants who dropped out or provided fewer than three responses were excluded. Second, participants who received warnings for three consecutive days due to low conscientiousness in completing the EMA measures were excluded. Third, data from participants whose total daily response time was less than 25% of the average response time or whose self‐rating of conscientiousness was below 60 were treated as missing values for those days. Nonresponses or late responses were also treated as missing.

After data cleaning, simple correlations were calculated between time spent on each APP and AASMU. Three apps, Rednote, TikTok, and Kuaishou, showed the highest correlation with AASMU. Based on the literature review, these apps were classified as highly visual social media. Consequently, we summed the time spent on these three apps to obtain the total daily time spent on social media in minutes.

The analysis was conducted in Mplus8.10 (Muthúen & Muthúen, 1998–2024). We used 10,000 MCMC iterations and two chains with random seeds. Default priors of Mplus were used for Bayesian estimation. Missing data were handled using the Kalman filter method implemented via the TINTERVAL option in Mplus [54]. The Kalman filter is a recursive algorithm based on hidden Markov models that uses information of prior time point to predict missing data and holds predictions when data are unavailable [55, 56]. This approach is particularly well‐suited for ILD with occasional missingness as it preserves the time‐series structure without requiring listwise deletion or multiple imputation. We applied the default convergence criterion in Mplus: a potential scale reduction PSR < 1.1 for each parameter [54]. The Mplus output files for the analyses are available in the Supporting Information “Model 1 in Study 1_output.txt,” “Model 2 in Study 1_output.txt.”

### 3.2. Results

During the 20‐day experiment, 31 subjects dropped out, resulting in a total of 206 subjects who completed Study 1. No subjects were excluded through data cleaning process, so the final sample consisted of 206 valid participants (see Figure 3). The overall percentage of missing data was 0.7%. Note that 177 participants achieved a 100% completion rate (overall completion rate = total number of missing measurements / number of subjects  ^∗^ number of observations). To ensure data integrity in Study 1, we screened for duplicate entries, careless responding (i.e., total response time less than 25% of the individual mean), and univariate outliers in key variables (i.e., AASMU or SMU scores exceeding ±3 SDs from the individual mean). No duplicate cases, careless responses, or univariate outliers were identified following these procedures. The original data can be found in the Supporting Information “Study 1 data.dat.”

Following Asparouhov et al. [43], we calculated the correlation between the DSEM‐estimated and observed person‐level means. The correlations for AASMU and SMU were 0.969 and 0.996 for Model 1 and Model 2, suggesting an excellent correspondence between model‐predicted and actual values, thus indicating a good model fit. The estimates for the fixed parameters and their 95% credible intervals (CIs) are shown in Table 1 (Model 1) and Table S2 (Model 2). In the Bayesian framework, a parameter is considered statistically significant if its 95% CI does not include zero, analogous to a *p*‐value <0.05 in frequentist inference. Results of Model 1 indicated that AASMU exhibited significant temporal stability across days, indicating that individuals’ anxiety levels tended to persist over time (*γ*_10_ = 0.36, 95% CI = [0.09, 0.62]). However, the autoregressive effect of SMU was not significant (*γ*_40_ = 0.17, 95% CI = [−0.06, 0.41]). Regarding time effects, AASMU showed a small but significant decrease over the 20‐day period (*γ*_50_ = −0.41, 95% CI = [−0.75, −0.07]), whereas SMU remained stable (*γ*_60_ = 0.02, 95% CI = [−0.06, 0.10]). The cross‐lagged associations between AASMU and SMU were near zero, providing no evidence of delayed reciprocal effects across days (*γ*_20_ = 0.00, 95% CI = [−0.03, 0.03], *γ*_30_ = 0.00, 95% CI = [−0.07, 0.08]). A significant positive covariance was observed between the person‐specific innovation terms of AASMU and SMU (*γ*_90_ = 0.24, 95% CI = [0.02, 0.45]). This indicates that, after controlling for autoregressive and cross‐lagged effects, the residuals of AASMU and SMU remained positively correlated within the same day. The results for AASMU and SMU in Model 2 were similar to Model 1 (see Table S3). It should be noted that all parameters were estimated while controlling for SEC or HL.

**Table 1:** Results of Model 1 in the EMA study.

Parameters		Unstandardized estimates			Standardized estimates		
		*b*	SD	95% CI	*β*	SD	95% CI
Fixed effect	${\text{AASMU}}_{i.t-1}^{\left(w\right)}$→${\text{AASMU}}_{i.t}^{\left(w\right)}\left(\gamma_{10}\right)$	**0.36**	**0.14**	**[0.01, 0.62]**	**1.45**	**0.56**	**[0.37, 2.55]**
	${\text{SMU}}_{i.t-1}^{\left(w\right)}$→${\text{SMU}}_{i.t}^{\left(w\right)}\left(\gamma_{40}\right)$	0.17	0.12	[−0.06, 0.41]	0.85	0.59	[−0.29, 2.03]
	${\text{SMU}}_{i.t-1}^{\left(w\right)}$→${\text{AASMU}}_{i.t}^{\left(w\right)}\left(\gamma_{20}\right)$	0.00	0.01	[−0.03, 0.03]	0.11	0.56	[−1.00, 1.20]
	${\text{AASMU}}_{i.t-1}^{\left(w\right)}$→${\text{SMU}}_{i.t}^{\left(w\right)}\left(\gamma_{30}\right)$	0.00	0.04	[−0.07, 0.08]	0.11	1.39	[−2.55, 2.89]
	**Time→** ${\text{AASMU}}_{i.t}^{\left(w\right)}\left(\gamma_{50}\right)$	**−0.41**	**0.18**	**[−0.75, −0.07]**	**−1.16**	**0.50**	**[−2.14, −0.19]**
	Time→${\text{SMU}}_{i.t}^{\left(w\right)}\left(\gamma_{60}\right)$	0.02	0.04	[−0.06, 0.01]	0.80	1.38	[−2.02, 3.34]
	${\mathrm{AASMU}}_{i.t}^{\left(w\right)}$ **↔** ${\mathrm{SMU}}_{i.t}^{\left(w\right)}\left(\gamma_{90}\right)$	**0.24**	**0.11**	**[0.02, 0.45]**	**2.57**	**1.38**	**[0.26, 5.90]**
	**Residual variance of AASMU** (*γ*_70_ **)**	**4.85**	**0.42**	**[4.02, 5.67]**	**4.67**	**0.45**	**[3.77, 5.53]**
	**Residual variance of SMU** (*γ*_80_)	**4.23**	**1.62**	**[1.03, 7.35]**	**1.00**	**0.39**	**[0.24, 1.74]**
	**SC→** *μ*_**AASMU**.**i**_ **(** *γ*_010_ **)**	**8.40**	**2.08**	**[4.43, 12.53]**	**0.21**	**0.05**	**[0.11, 0.30]**
	HL→*μ*_AASMU.*i*_(*γ*_011_)	−1.91	3.71	[−8.33, 6.10]	−0.02	0.07	[−0.15, 0.11]
	**SEC→** *μ*_AASMU.*i*_ **(** *γ*_012_ **)**	**−6.84**	**2.74**	**[−12.13, −1.36]**	**−0.17**	**0.07**	**[−0.30, −0.04]**
	SEC→*μ*_SMU.*i*_(*γ*_021_)	−19.70	12.68	[−44.69, 4.98]	−0.08	0.05	[−0.18, 0.02]
	SEC→*ϕ*_11.*i*_ (*γ*_11_)	−0.02	0.04	[−0.10, 0.06]	−0.03	0.07	[−0.17, 0.10]
	SEC→*ϕ*_22.*i*_ (*γ*_41_)	0.01	0.04	[−0.06, 0.08]	0.03	0.08	[−0.13, 0.18]
	SEC→*ϕ*_12.*i*_ (*γ*_21_)	0.00	0.00	[−0.01, 0.01]	−0.01	0.07	[−0.15, 0.13]
	SEC→*ϕ*_21.*i*_ (*γ*_31_)	0.00	0.01	[−0.03, 0.02]	−0.03	0.20	[−0.43, 0.36]
	SEC→*ϕ*_13.*i*_ (*γ*_51_)	0.07	0.05	[−0.03, 0.17]	0.08	0.06	[−0.04, 0.20]
	SEC →*ϕ*_23.*i*_ (*γ*_61_)	−0.01	0.01	[−0.03, 0.02]	−0.12	0.20	[−0.49, 0.29]
	**SEC→**log(*π*_1.**i**_)**(** *γ*_71_ **)**	**−0.39**	**0.13**	**[−0.65, −0.14]**	**−0.16**	**0.05**	**[−0.26, −0.06]**
	SEC→log(*π*_2.*i*_)(*γ*_81_)	0.90	0.50	[−0.07, 1.90]	0.09	0.05	[−0.01 0.18]
	**SEC→**cov_1.**i**_ **(** *γ*_91_ **)**	**−0.09**	**0.03**	**[−0.15, −0.02]**	**−0.40**	**0.19**	**[−0.86, −0.10]**

Residual variance	**AASMU (** **u**_01**i**_ **)**	**253.27**	**28.42**	**[203.66, 316.37]**	**0.92**	**0.03**	**[0.86, 0.96]**
	**SMU (** **u**_02**i**_ **)**	**1076.12**	**1236.10**	**[8843.21, 13717.69]**	**0.99**	**0.01**	**[0.97, 1.00]**
	${\mathrm{AASMU}}_{i.t-1}^{\left(w\right)}$ **→** ${\mathrm{AASMU}}_{i.t}^{\left(w\right)}\left(u_{11i}\right)$	**0.06**	**0.01**	**[0.04, 0.09]**	**1.00**	**0.01**	**[0.97, 1.00]**
	${\mathrm{SMU}}_{i.t-1}^{\left(w\right)}$ **→** ${\mathrm{SMU}}_{i.t}^{\left(w\right)}\left(u_{22i}\right)$	**0.04**	**0.01**	**[0.03, 0.06]**	**1.00**	**0.01**	**[0.97, 1.00]**
	${\mathrm{SMU}}_{i.t-1}^{\left(w\right)}$ **→** ${\mathrm{AASMU}}_{i.t}^{\left(w\right)}\left(u_{12i}\right)$	**0.00**	**0.00**	**[0.00, 0.00]**	**1.00**	**0.01**	**[0.97, 1.00]**
	${\mathrm{AASMU}}_{i.t-1}^{\left(w\right)}$ **→** ${\mathrm{SMU}}_{i.t}^{\left(w\right)}\left(u_{21i}\right)$	**0.00**	**0.00**	**[0.00, 0.00]**	**0.98**	**0.06**	**[0.80, 1.00]**
	**Time→** ${\mathrm{AASMU}}_{i.t}^{\left(w\right)}\left(u_{13i}\right)$	**0.12**	**0.03**	**[0.08, 0.18]**	**0.99**	**0.01**	**[0.96, 1.00]**
	**Time→** ${\mathrm{SMU}}_{i.t}^{\left(w\right)}\left(u_{23i}\right)$	**0.00**	**0.00**	**[0.00, 0.00]**	**0.97**	**0.07**	**[0.75, 1.00]**
	${\mathrm{AASMU}}_{i.t}^{\left(w\right)}$ **↔** ${\mathrm{SMU}}_{i.t}^{\left(w\right)}\left(u_{\mathrm{cov}.i}\right)$	**0.01**	**0.01**	**[0.00, 0.02]**	**0.84**	**0.18**	**[0.26, 0.99]**
	**Residual variance of AASMU (** **u**_*π*1.**i**_ **)**	**1.05**	**0.13**	**[0.83, 1.34]**	**0.98**	**0.02**	**[0.94, 1.00]**
	**Residual variance of SMU (** **u**_*π*2.**i**_ **)**	**17.88**	**1.79**	**[14.73, 21.74]**	**0.99**	**0.01**	**[0.97, 1.00]**

*Note:* Unstandardized estimations were recommended because many cases were removed by Mplus when calculating standardized estimations. The significant estimates were highlighted with boldface.

Abbreviations: SD, standard deviation of posterior distribution; 95% CI, 95% credible interval.

For the covariates, social comparison emerged as a significant positive predictor of AASMU (*γ*_010_ = 8.40, 95% CI = [4.43, 12.53] in Model 1; *γ*_010_ = 8.43, 95% CI = [4.46, 12.55] in Model 2), suggesting that individuals with a higher tendency for social comparison experienced greater appearance anxiety. In contrast, SC had a negative effect on AASMU (*γ*_012_ = −6.84, 95% CI = [−12.13, −1.36] in Model 1; *γ*_012_ = −5.70, 95% CI = [−10.86, −0.40] in Model 2), indicating that more self‐compassionate individuals experienced lower appearance anxiety. Beyond mean‐level effects, both SC and HL were negatively related to the residual variance of AASMU (*γ*_71_ = −0.40, 95% CI = [−0.65, −0.14] in Model 1; *γ*_71_ = −0.42, 95% CI = [−0.78, −0.05] in Model 2), suggesting that individuals high in these protective traits displayed more stable day‐to‐day emotional patterns with less fluctuation in appearance anxiety. Furthermore, these two factors also attenuated the same‐day association between AASMU and SMU (*γ*_91_ = −0.09, 95% CI = [−0.15, −0.02] in Model 1; *γ*_91_ = −0.14, 95% CI = [−0.23, −0.04] in Model 2), implying that individuals with greater SC or healthier lifestyles were less prone to experience concurrent increases in appearance anxiety alongside SMU. Finally, all random effects were significant, reflecting substantial individual differences across these coefficients.

## 4. Study 2: The EMI Study

### 4.1. Statistical Analysis

In Study 2, a DSEM was applied to data from a randomized control trial design with pre–post tests. First, as Study 1 revealed only a concurrent effect between the residuals of AASMU and SMU, and the autoregressive effect of SMU was not significant, we assumed that SMU acted as a time‐varying variable with a concurrent effect on AASMU, which was consistent with previous studies [57]. Second, Study 1 found that SC and SEC had significant effects on the means of AASMU, while SEC and HL negatively predicted intra‐individual variation in AASMU. Therefore, we included these three variables as control variables in Study 2. In summary, we focused on a pretest–post‐test DSEM with AASMU as the outcome, SMU as its concurrent predictor, and HL, SC, and SEC as the predictors of AASMU at the between‐person level, with SEC and HL also predicting the residuals of AASMU.

The formulas for the hypothesized model are shown in Equations (15)–(32), where the subscripts *pre* and *post* represent the pretest and post‐test, respectively, and *G*_*i*_ represents the group (i.e., intervention or control group). All other variables and symbols remain the same as in Study 1. For example, ${\text{AASMU}}_{\text{it,pre}}^{\left(w\right)}$ denotes AASMU of the *i*th subject at time *t* during the pretest period.

#### 4.1.1. Pretest Period

Within‐person level model:

(15) AASMUit,prew=ϕ11i,preAASMUit−1,prew+ϕ12i,preSMUit,prew+ϕ13i,preTimepre+δ1it,pre,δ1it,pre∼N0,π1i,pre,

(16) SMUit,prew=ϕ14i,preTimepre+δ2it,pre,δ2it,pre∼N0,π2i,pre.

Between‐person level model:

(17) AASMUi,preb=γ01.pre+γ010.preGi+u01i.pre,

(18) SMUit,preb=γ02.pre+γ020.preGi+u02i.pre,

(19) ϕ11i,pre=γ10.pre+γ11.preGi+u11i.pre,

(20) ϕ12i,pre=γ20.pre+γ21.preGi+u12i.pre,

(21) ϕ13i,pre=γ30.pre+γ31.preG+u13i.pre,

(22) ϕ14i,pre=γ40.pre+γ41.preGi+u14i.pre,

(23) logπ1i,pre=γ50.pre+γ51.preGi+uπ1i.pre,

(24) logπ2i,pre=γ60.pre+uπ2i.pre.

#### 4.1.2. Intervention Period

Within‐person level model:

(25) AASMUit,postw=ϕ11i,postAASMUit−1,postw+ϕ12i,postSMUit,postw+ϕ13i,postTimepost+δ1it,post,δ1it,post∼N0,π1i,post,

(26) SMUit,postw=ϕ14i,postTimepost+δ2it,post,δ2it,post∼N0,π2i,post.

Between‐person level model:

(27) ΔAASMUib=γ01.change+γ010.changeGi+γ011.changeHLi+γ012.changeSCi+γ013.changeSECi+u01i.change,

(28) ΔSMUi,tb=γ02.change+γ020.changeGi+u02i.change,

(29) Δϕ11,i=γ10.change+γ11.changeGi+u11i.change,

(30) Δϕ12,i=γ20.change+γ21.changeGi+u12i.change,

(31) Δϕ13,i=γ30.change+γ31.changeGi+u13i.change,

(32) Δϕ14,i=γ40.change+γ41.changeGi+u14i.change,

(33) Δlogπ1i=γ50.change+γ51.changeGi+γ52.changeSECi+γ53.changeHLi+uπ1i.change.

There are three concerns when using DSEM to analyze pretest–post‐test data: baseline differences, pretest–post‐test time effects, and intervention effects. For the baseline differences, the regression coefficients of the group variable at the between‐person level in pretest stage assess whether there are differences between the intervention and control groups before the intervention Equations (17)–(24). For the pretest–post‐test time effects, the intercepts in Equations (27)–(33) capture changes in the control group while holding the time‐invariant covariates at their ground‐means. If these parameters differ from zero, they indicate a change (on average) due to time. For the intervention effects, the regression coefficients for the dummy variable *G*_*i*_ (i.e., *γ*_010.change_, *γ*_020.change_, *γ*_11.change_, *γ*_21.change_, *γ*_31.change_, *γ*_41.change_, and *γ*_51.change_) capture differential changes between the two groups when time‐invariant covariates are held constant at their ground‐means. Hence, if these coefficients differ from zero, it signifies an intervention effect.

The data cleaning procedure for Study 2 was similar to that of Study 1, except that participants were classified as inattentive respondents based on their self‐rated conscientiousness regarding reading material as well. The data were analyzed using the same default settings in Mplus as in Study 1, with the same number of iterations and number of chains. The Mplus output file of the analyses can be found in the Supporting Information “Study 2_output.txt.”

### 4.2. Results

Six subjects withdrew from the experiment during the last 20 days. Consequently, the final sample comprised 200 participants who completed the full 40‐day study (105 in the intervention group and 95 in the control group). The overall percentage of missing data for Study 2 was 10.7%, with 142 participants achieving a 100% completion rate. The average conscientiousness scores for all participants during the last 20 days were all above 60, so no one was excluded from the data, resulting in a total of 200 valid participants. We applied the same data screening procedures as in Study 1. No outliers were detected after data cleaning. The original data can be downloaded from the Supporting Information “Study 2 data.dat.” The correlations between estimated and observed individual means for AASMU at the pretest stage and the post‐test stage were 0.988 and 0.985, respectively, supporting the adequacy of model fit under the DSEM framework.

#### 4.2.1. Initial Differences

At the pretest stage, no significant differences were observed between the intervention and control groups across any baseline parameters (Table 2). Specifically, there were no differences in the mean levels of AASMU (*γ*_010.pre_ = 0.45, 95% CI = [−4.02, 5.18]) or SMU (*γ*_020.pre_ = −3.14, 95% CI = [−34.06, 28.99]). Likewise, the two groups did not differ in the autoregressive effect of AASMU (*γ*_11.pre_ = 0.09, 95% CI = [−0.00, 0.19]), the effect of SMU on AASMU (*γ*_21.pre_ = 0.00, 95% CI = [−0.01, 0.01]), or the time effect on AASMU (*γ*_31.pre_ = 0. 08, 95% CI = [−0.02, 0.18]) and SMU (*γ*_41.pre_ = −0.80, 95% CI = [−2.06, 0.53]). Moreover, the residual variance of AASMU did not differ across groups (*γ*_51.pre_ = 0.09, 95% CI = [−0.00, 0.19]). Taken together, these results confirm that participants were successfully randomized and that the two groups were statistically equivalent at baseline in both mean levels and dynamic parameters.

**Table 2:** Results of the EMI study.

Effects	Parameters	Unstandardized estimates			Standardized estimates		
		*b*	SD	95% CI	*β*	SD	95% CI
Initial differences	*γ*_010.pre_	0.45	2.39	[−4.02, 5.18]	0.01	0.05	[−0.09, 0.01]
	*γ*_020.pre_	−3.14	15.81	[−34.06, 28.99]	−0.01	0.05	[−0.11, 0.09]
	*γ*_11.pre_	0.09	0.05	[−0.00, 0.16]	0.15	0.08	[−0.00, 0.31]
	*γ*_21.pre_	0.00	0.01	[−0.01, 0.01]	0.04	0.07	[−0.10, 0.18]
	*γ*_31.pre_	0.08	0.05	[−0.02, 0.18]	0.14	0.09	[−0.03, 0.31]
	*γ*_41.pre_	−0.80	0.67	[−2.06, 0.53]	−0.08	0.07	[−0.21, 0.05]
	*γ*_51.pre_	0.09	0.05	[−0.00, 0.19]	0.15	0.08	[−0.00, 0.31]
Time effect	*γ*_01.change_	−0.92	6.76	[−14.16, 12.48]	−0.05	0.38	[−0.80, 0.71]
	*γ*_02.change_	2.19	5.51	[−8.92, 13.19]	0.02	0.05	[−0.08, 0.10]
	*γ*_10.**change**_	**−0.17**	**0.05**	**[−0.25, −0.08]**	**−0.55**	**0.16**	**[−0.89, −0.27]**
	*γ*_20.change_	0.01	0.00	[−0.00, 0.01]	0.14	0.12	[−0.10, 0.38]
	*γ*_30.**change**_	**0.12**	**0.03**	**[0.08, 0.18]**	**0.53**	**0.13**	**[0.28, 0.79]**
	*γ*_40.change_	−1.20	0.74	[−2.67, 0.27]	−0.18	0.11	[−0.40, 0.04]
	*γ*_50.**change**_	**−0.16**	**0.05**	**[−0.25, −0.07]**	**−0.53**	**0.16**	**[−0.85, −0.22]**
Intervention effect	*γ*_010.**change**_	**−2.72**	**1.17**	**[−4.96, −0.42]**	**−0.05**	**0.02**	**[−0.10, −0.01]**
	*γ*_020.change_	−2.67	7.73	[−17.85, 12.43]	−0.01	0.02	[−0.05, 0.04]
	*γ*_11.change_	−0.02	0.06	[−0.14, 0.10]	−0.03	0.07	[−0.17, 0.11]
	*γ*_21.change_	0.00	0.01	[−0.01, 0.01]	0.00	0.06	[−0.12, 0.11]
	*γ*_31.**change**_	**−0.31**	**0.07**	**[−0.45, −0.17]**	**−0.26**	**0.06**	**[−0.38, −0.14]**
	*γ*_41.change_	1.34	1.03	[−0.69, 3.39]	0.07	0.06	[−0.04, 0.18]
	*γ*_51.**change**_	**−0.48**	**0.17**	**[−0.82, −0.16]**	**−0.11**	**0.04**	**[−0.19, −0.04]**

*Note:* Unstandardized estimations were reported because many cases were removed by Mplus when calculating standardized estimations. The significant estimates were highlighted with boldface.

Abbreviations: SD, standard deviation of posterior distribution; 95% CI, 95% credible interval.

#### 4.2.2. Pretest–Post‐Test Time Effect

Across the pretest–post‐test period, no significant changes were observed in the mean levels of AASMU (*γ*_01.change_ = −0.92, 95% CI = [−14.16, 12.48]) or SMU (*γ*_02.change_ = 2.19, 95% CI = [−8.92, 13.19]), nor in the effect of SMU on AASMU (*γ*_20.change_ = 0.01, 95% CI = [−0.00, 0.01]), or the time effect on SMU (*γ*_40.change_ = −1.20, 95% CI = [−2.67, 0.27]). However, the autoregressive effect of AASMU significantly decreased from pretest to post‐test (*γ*_10.change_ = −0.17, 95% CI = [−0.25, −0.08]) was negatively significant, suggesting that the inertia of AASMU became weaker over time in the control group. The time effect of AASMU was also positively significant (*γ*_30.change_ = 0.12, 95% CI = [0.08, 0.18]). Given that a negative time trend was observed during the pretest phase for the control group (*γ*_30.pre_ = −0.22, 95% CI = [−0.30, −0.15]), this suggests that the rate of anxiety reduction slowed following the intervention period for the control group. Finally, the fixed intercept for the change in the log of the residual variance of AASMU was significant (*γ*_50.change_ = −0.16, 95% CI = [−0.25, −0.07]), reflecting a decline in within‐person variability of appearance anxiety from pretest to post‐test in the control group.

#### 4.2.3. Intervention Effects

The intervention produced several significant effects on appearance anxiety. First, the mean level of AASMU decreased significantly more in the intervention group than in the control group (*γ*_010.change_ = −2.72, 95% CI = [−4.96, −0.43]), demonstrating a clear intervention effect. Second, the time slope of AASMU also showed a significant group difference (*γ*_31.change_ = −0.30, 95% CI = [−0.45, −0.17]), indicating that the intervention group experienced a *faster decline* in appearance anxiety over time. Third, the residual variance of AASMU was significantly reduced (*γ*_51.change_ = −0.48, 95% CI = [−0.82, −0.16]), suggesting that fluctuations in appearance anxiety became more stable following the intervention. In contrast, no significant intervention effects were found for the autoregressive effect of AASMU (γ_11_ = −0.02, 95% CI = [−0.14, 0.10]), the concurrent effect of SMU on AASMU (γ_21_ = 0.00, 95% CI = [−0.01, 0.01]), the time effect on SMU (γ_41_ = 1.34, 95% CI = [−0.69, 3.39]), or the mean of SMU (γ_020_ = −2.67, 95% CI = [−17.85, 12.43]). Together, these results indicate that the SC–based EMI effectively reduced both the average level and IIV of AASMU.

**Figure 5:**
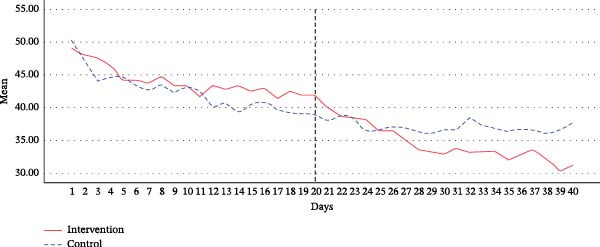
Trends in mean scores of AASMU about physical appearance status in the intervention group and control group. Note: This time‐series plot is based on the average AASMU scores across all participants in both the intervention and control groups.

To describe the trajectory of AASMU, we averaged AASMU for all participants in both the intervention group and control group, respectively, on each day and drew a time series plot. The *x*‐axis represents days, while the *y*‐axis represents the mean value of AASMU, with a dotted vertical line indicating the day the intervention began. As illustrated in Figure 5, AASMU in the intervention group declined significantly after the intervention started (day 21), immediately after the start of the intervention after day 20. This rapid decline continued until day 29, after which the rate of decline leveled off. By days 30–40 (the midpoint of the intervention), AASMU stabilized within a certain range of 30–35. In contrast, the control group showed little to no decline, with AASMU remaining between 35 and 40 by the end. These findings suggested that self‐compassionate statements are effective in reducing AASMU, with the reduction remaining stable over time.

## 5. Discussion and Conclusion

**Figure 6:**
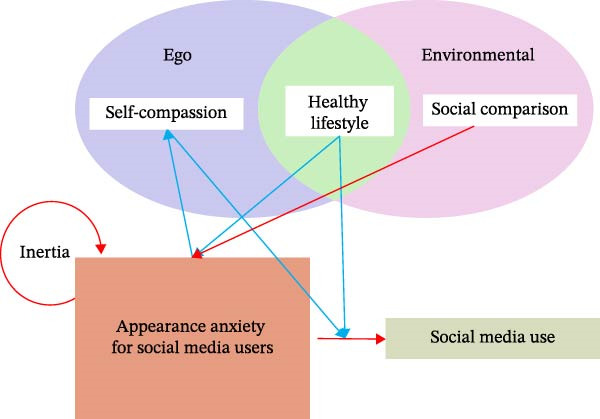
Rationale model.

In Study 1, we examined the dynamic process and relationship between AASMU and SMU using an EMA design within a DSEM framework. Our findings align with and extend both objectification theory and sociocultural theory (see Figure 6).

At the within‐person level, AASMU showed a significant positive autoregressive effect, suggesting that individuals’ appearance anxiety tends to remain stable across time. This may be consistent with objectification theory [3], individuals who internalize an observer’s perspective often engage in habitual body monitoring and self‐surveillance. These cognitive‐emotional patterns are likely to persist, resulting in emotional inertia. AASMU and SMU also showed a dynamic and contemporaneous association of residuals on the same day, suggesting that AASMU is a state‐like emotional response sensitive to daily fluctuations. This finding is consistent with recent EMA evidence indicating that momentary changes in emotion regulation can predict and result from social media engagement, forming a short‐term bidirectional interplay between emotional states and online behaviors [16]. It also implies that timely interventions, such as the EMI implemented in Study 2, could be particularly effective in reducing AASMU.

However, we did not observe significant cross‐lagged effects between AASMU and SMU. One possible explanation is the daily measurement interval used in the current study may not have been sufficiently frequent to capture the hypothesized bidirectional dynamics between the two constructs. Prior research suggests that the optimal sampling frequency in EMA should align with the fluctuation rate of the target variables, and several studies have employed more intensive schedules (e.g., six beeps per day) to detect emotion‐related dynamics (e.g., [58]). Other factors, such as model misspecification or contextual influences, may also contribute. At the same time, the null finding may indicate a genuine absence of causal influence, rather than methodological limitations alone. The strong contemporaneous residual association observed further suggests that short‐term dynamics may overshadow lagged effects in daily data. In addition, the non‐significant autoregressive effect of SMU may reflect the situational and externally driven nature of SMU. It also suggests that SMU may be less temporally stable than affective states like AASMU. This aligns with the idea that SMU is strongly shaped by daily context, routine variability, and external demands, rather than internal psychological continuity.

At the between‐person level, predictors of AASMU can be grouped into two domains: individual differences and social‐environmental factors. From the ego perspective, consistent with previous research, higher SC was associated with lower AASMU [59]. Based on objectification theory, SC may reduce self‐judgment and vulnerability to anxiety triggered by self‐objectification. Moreover, SC enhanced emotional stability in AASMU and reduced daily co‐fluctuations between AASMU and SMU. SC may serve as a regulatory buffer that interrupts the reinforcing loop between online behaviors and negative affect, helping individuals maintain more stable emotional states across time.

From the social‐environmental perspective, individuals with a greater tendency for social comparison tended to report higher AASMU. This is consistent with sociocultural theory, which posits that internalization of societal appearance ideals (often perpetuated by social media) leads to self‐evaluation against unrealistic standards. Upward social comparisons are strongly associated with negative emotions, body dissatisfaction, reduced self‐esteem, and disordered eating behaviors [60]. In collectivist cultures such as China, social comparison is deeply embedded in social norms, which may intensify appearance‐related pressures in digital environments. Our findings support social comparison as a key sociocultural mechanism driving appearance anxiety. Additionally, a HL reduced the positive same‐day correlation between the residuals of AASMU and SMU and lowered the IIV of AASMU. A HL involves not only individual health behaviors but also positive social interactions [12]. From the perspective of sociocultural theory, a HL may buffer against the internalization of beauty standards by promoting self‐regulation and a balanced self‐concept [61]. Therefore, encouraging a HL may reduce susceptibility to social media–induced appearance anxiety and serve as a protective factor in digital contexts.

Based on Study 1, Study 2 examined the feasibility and potential benefits of reading SC statements as an intervention to reduce AASMU. Participants in the intervention group showed a more pronounced and accelerated decrease in AASMU compared with the control group, consistent with cumulative effects of EMI. This finding aligns with previous evidence supporting the usefulness of EMI in everyday contexts [39, 62]. In addition, the reduction in IIV of AASMU has important implications for mental health. Previous research indicates that elevated IIV is linked to mood instability, attentional difficulties, and cognitive dysfunction and is increasingly recognized as a marker of general risk for psychopathology and emotion dysregulation [63, 64]. At the same time, IIV has been considered a meaningful intervention outcome, reflecting changes in emotional regulation and neural processing consistency [35, 65]. Thus, our finding suggests that the EMI alleviates momentary distress and is consistent with promoting greater emotional consistency, a hallmark of adaptive functioning and psychological resilience. While these results may support interpretations involving improved emotion regulation.

The success of the SC intervention provides important theoretical insights. For one thing, SC fosters a non‐judgmental and accepting attitude toward one’s body, thereby mitigating the emotional consequences of self‐objectification and serving as a psychological buffer against internalized surveillance [59]. For another, consistent with sociocultural theory, SC appears to protect individuals from the negative impact of upward social comparisons commonly triggered by appearance‐focused content on social media [11]. This interpretation is also supported by findings from Study 1, which showed that individuals with higher levels of SC exhibited weaker same‐day co‐fluctuations between AASMU and SMU and more stable appearance anxiety. Theoretically, these results support the role of SC as a dynamic regulatory mechanism that can interrupt socioculturally reinforced appearance‐related stress in real time.

Based on the current findings, we propose several practical recommendations for implementing SC interventions in real‐world settings. First, the same‐day contemporaneous associations of residuals between AASMU and SMU highlight the need for timely support. We recommend delivering EMI prompts on days when individuals show signs of excessive social media engagement—via mobile notifications or passive tracking systems. EMI tools can be designed to detect spikes in screen time and trigger brief, supportive SC prompts in real time. Second, given the efficacy of SC intervention for AASMU, app‐based EMI programs could offer daily evidence‐based prompts (e.g., self‐kindness reminders and compassionate reframing exercises), with options for personalization and short‐term goals to enhance adherence. Last, SC content can be embedded into existing digital mental health infrastructures, such as school‐based programs, workplace wellness initiatives, or online therapy platforms.

The current study has several limitations. First, most variables were assessed using self‐report instruments, which are subject to common sources of bias, such as social desirability. Although SMU was measured via screenshot‐based recording, device‐specific inconsistencies may still exist. Future studies could consider multimodal assessment strategies, including physiological indicators (e.g., heart rate variability, skin conductance) and more direct objective behavioral tracking methods (e.g., app usage data via tracking software). Second, as nonsignificant cross‐lagged effects were found between AASMU and SMU, future studies may use more frequent EMA prompts, explore alternative DSEM specifications, increase sample size and number of time points, or better control contextual factors to clarify whether the null cross‐lagged effects reflect a true absence of dynamic relationship or methodological limitations. Third, this study recruited only Chinese female participants. While justified by the high prevalence of AASMU among women, this limits generalizability. Future work should examine whether findings extend across genders and cultural contexts. Additionally, excluding 28 “careless” respondents prior to randomization ensured data quality but may have slightly reduced representativeness, as excluded individuals could differ systematically (e.g., younger, more digitally fluent). Fourth, the intervention was fixed and non‐adaptive. Future studies could enhance personalization by developing just‐in‐time adaptive interventions. For instance, participants could first rate which SC statements they find most effective. AI‐assisted systems could then generate similar tailored content and deliver it dynamically based on each individual’s emotional state. Last, this study did not include a follow‐up period to assess the long‐term sustainability of intervention effects. With the development of hierarchical autoregressive growth models [66], future studies should incorporate follow‐up assessments to evaluate durability in multi‐stage EMI designs and adopt stricter designs in which randomization occurs before the EMA phase, thereby reducing risks of reactivity, baseline drift, or chance trajectory differences that could bias intervention outcomes.

In summary, the EMA supports a contemporaneous relationship between SMU and appearance anxiety. Additionally, a higher level of SC or a healthier lifestyle moderate this relationship, providing resilience against the negative effects of SMU. Individuals with a greater tendency for social comparison or lower levels of SC are likely to experience more appearance anxiety. The EMI study indicates that the intervention based on reading SC statements is an effective intervention to reduce both the mean level and intra‐individual variability of appearance anxiety. These results highlight the potential of EMI as a practical and scalable approach for addressing appearance anxiety in everyday life.

## Author Contributions

Yue Liu was responsible for the study’s design and data analysis.

## Funding

This study was supported by The Youth Talent Project of the Philosophy and Social Science Foundation of Sichuan Province (SCJJ25QN17).

## Conflicts of Interest

The authors declare no conflicts of interest.

## Supporting Information

Additional supporting information can be found online in the Supporting Information section.

## Supporting information


**Supporting Information** The supporting materials include: Text S1: Scale development and validation procedures for AASMU. Supporting Text S2: CFA Results for SC, HL, and SEC. Text S3: The procedure for developing reading materials used in EMI. Text S4: Reading materials in EMI. Table S1: Power, relative bias, and width of credible interval of the fixed effect of the cross‐lagged parameters. Table S2: Power, relative bias, and width of credible interval of the fixed intervention effect on the mean, autoregressive effect, and intra‐individual variance based on a random‐group design. Table S3: Results of Model 2 in the EMA study. Figure S1: Example screenshot of daily screen usage time. Figure S2: Analytical model of the dynamic relationship between the different levels of each variable in Model 2.

## Data Availability

The data that supports the findings of this study are available in the supporting material of this article.
