# Supplementary material for: Understanding and Reducing Appearance Anxiety for Social Media Users: A Study Using Ecological Momentary Assessment and Ecological Momentary Intervention
Source: Depress Anxiety. 2026 Jun 15;2026:7212945. doi: 10.1155/da/7212945 (PMC13266371; doi:10.1155/da/7212945)
Supplement: Supplementary file 1 — Supporting Information The supporting materials include: Text S1: Scale development and validation procedures for AASMU. Supporting Text S2: CFA Results for SC, HL, and SEC. Text S3: The procedure for developing reading materials used in EMI. Text S4: Reading materials in EMI. Table S1: Power, relative bias, and width of credible interval of the fixed effect of the cross‐lagged parameters. Table S2: Power, relative bias, and width of credible interval of the fixed intervention effect on the mean, autoregressive effect, and intra‐individual variance based on a random‐group design. Table S3: Results of Model 2 in the EMA study. Figure S1: Example screenshot of daily screen usage time. Figure S2: Analytical model of the dynamic relationship between the different levels of each variable in Model 2. [file DA-2026-7212945-s001.zip › supplementary materials-5.19.docx]

**Scale Development and Validation Procedures for AASMU**

We developed the full version of AASMU, following strict psychometric standards in three distinct phases: initial scale development, refinement, and measurement validation. Sample 1 (*N*=877) was used for item development and exploratory factor analyses. Sample 2 (*N*=608) was used to assess internal consistency reliability, validity, and measurement invariance across genders. Sample 3 (*N*=228) was retested after a 2-month interval to evaluate retest reliability. In addition, the Social Appearance Anxiety Scale (SAAS) and the self-consciousness subscale of the Chinese Social Anxiety Scale (SAC) were used to assess convergent validity. The results indicated that AASMU comprised two dimensions: anxiety experience (e.g., “I'd be nervous about taking a photo that would be posted on social media,” with a total of 10 items) and body monitoring (e.g., “I'll check my social media posts about my appearance,” with a total of 7 items). Items were rated on a 5-point scale (1=Not at all to 5=Absolutely) and summed to produce an index of AASMU. Higher scores indicate a higher level of appearance anxiety, while low scores reflected a lower level. The internal consistency and retest reliabilities of the scale reached 0.90 and 0.98 respectively. The between-person and within-person reliabilities for AASMU are 0.94 and 0.81 respectively (Geldhof et al., 2014). The two-factor model fitted fairly well based on sample 2 (i.e., χ²(*N*=608)=527.40, CFI=0.90, TLI =0.89, RMSEA=0.08, Hu & Bentler, 1999). Convergent validity was found to be 0.73 for SAAS and 0.61 for SAC indicating strong correlations. Measurement invariance revealed no gender differences for the scale scores. Overall, these findings supported AASMU as a psychometrically sound tool.

**The CFA Results for SC, HL, and SEC**

The SC scale demonstrated acceptable internal consistency (alpha=0.88) and retest reliability (0.89). For construct validity, the confirmatory factor analysis (CFA) revealed an acceptable fit of the two-factor model (i.e., χ² (*N* = 206) =14.60, CFI = 0.88, TLI = 0.93, RMSEA = 0.06). Moreover, a second-order factor model comprising two first-order factors (capability and perspective) fitted well (χ² (*N* = 206) = 68.58, CFI=0.96, TLI=0.95, RMSEA=0.05, SRMR=0.05).

The HL scale exhibited a Cronbach's alpha coefficient of 0.90, retest reliability of 0.71, and a good fit of the eight-factor model (i.e., χ2(*N* = 206) = 12.58, CFI = 0.93, TLI = 0.90, RMSEA = 0.04).Moreover, a second-order factor model comprising eight first-order factors (physical exercise behavior, regular lifestyle behavior, dietary nutrition behavior, health-risk behavior, health responsibility behavior, interpersonal support behavior, stress management behavior, and life appreciation behavior.) fitted well (χ² (*N* = 206)=841.37, CFI=0.91, TLI=0.90, RMSEA=0.04, SRMR=0.07).

The SEC scale demonstrated a Cronbach's alpha coefficient of 0.83 and a good fit of the six-factor model (i.e., χ² (*N* = 206) =16.25, CFI = 0.91, TLI = 0.91, RMSEA = 0.05). Moreover, a second-order factor model comprising six first-order factors (self-kindness, shared humanity, mindfulness, self-criticism, isolation, and over-identification) fitted well (χ² (*N* = 206) =459.87, CFI=0.91, TLI=0.90, RMSEA=0.05, SRMR=0.06).

**The Procedure for Developing Reading Materials Used in EMI**

The reading materials used in EMI were developed through the following process. First, a comprehensive literature review of intervention studies based on self-compassion theory was conducted. Sentences designed to enhance self-compassion were carefully selected from relevant studies, then adapted, translated, and reorganized to address our goal of reducing AASMU. All sentences used in the intervention group included three core dimensions: positive thinking, common humanity, and self-kindness. Second, neutral materials were developed through a similar process. Concepts related to physics, chemistry, and biology, as well as neutral prose free from emotional coloration, were selected, adapted, and restructured from secondary school textbooks. Initially, both the self-compassionate and neutral materials consisted of 30 statements, each containing 100 to 150 words.

Next, five graduate students majoring in the psychology of emotion evaluated the content validity of these sentences (e.g., assessing whether they accurately reflected the concepts of self-compassion or neutrality, whether they were fluent and coherent, grammatically correct, and free from ambiguity). Based on this evaluation, 10 statements with the highest content validity were chosen for each category of materials. The self-compassionate materials included sentences like: “I will remain calm and balanced when painful things happen,” “I accept painful experiences as part of life, everyone experiences pain,” “I will give myself the care and tenderness I need.”. The neutral materials included sentences such as: “In an ecosystem, animals are consumers of organic food. In the early development of life, when there were only cyanobacteria and bacteria on Earth, ecosystems were two-circuit systems of producers and decomposers.”

Finally, 28 female undergraduate students with no background in psychology and who had not participated in the formal experiment, rated each sentence based on its relevance to self-compassion and its perceived ability to relieve appearance anxiety. Ratings were based on a 9-point scale (1= Very irrelevant/no relief at all to 9= Very relevant/a great deal of relief). The results of paired sample t-tests revealed that self-compassionate statements were significantly more correlated with self-compassion than neutral statements (*t* = 4.923, *df* = 19, *p* < 0.001, *Cohen's d*=11.091). Furthermore, self-compassionate statements provided significantly greater perceived relief from appearance anxiety compared to neutral statements (*t* = 4.501, *df* = 19, *p* < 0.001, *Cohen's d* = 13.693). These results indicated that both types of materials were appropriate for use in the intervention and control groups of the EMI study.

**Reading Materials in EMI**

Self-compassion intervention materials:

(1) When painful events occur, I maintain calmness and view them with a balanced perspective; I regard painful experiences as an inevitable part of life that everyone encounters; I offer myself the care and tenderness I need. When others comment on my appearance, I calm my mind and respond objectively with balance; When I realize my appearance is imperfect, I tell myself: most people are imperfect; I maintain self-confidence and accept my physical flaws.

(2) When I feel frustrated, I attempt to calm my mind and balance my emotional experiences; I tell myself that life is difficult for everyone, I am not alone; I strive to be kind, accepting, understanding, and compassionate towards myself. When others comment on my appearance, I maintain an open, accepting, and balanced attitude; When I realize I have flaws in my appearance, I tell myself everyone has physical imperfections; I consistently treat my physical shortcomings with tolerance.

(3) When I realize I have made a mistake or failed, I try to view the situation dialectically from both positive and negative angles; I tell myself that everyone makes mistakes, experiences failure, and is imperfect; I forgive myself, analyze the issue rationally, and grant myself the necessary compassion. When I think about my physical flaws, I attempt to accept my feelings at that moment; When troubled by appearance issues, I feel I am not alone, others experience this too; I strive to be tolerant of my physical flaws and shortcomings.

(4) When feeling emotionally low, I approach myself with curiosity and openness, focusing on my present feelings, rarely suppressing or amplifying my emotions; I constantly remind myself: many people in the world feel the same way; I become more caring and loving towards myself. When I think about my physical flaws, I analyze them calmly rather than criticizing or denying them. When I feel I don't look very good, I remind myself that most people are dissatisfied with their own appearance. I try to understand and rationally address the aspects of my appearance I dislike.

(5) When something repeatedly troubles me, I consciously shift my attention to my physical and mental sensations, focusing more on my present feelings; I know most people experience troubling feelings, we all struggle in life; I wait patiently. When unpleasant memories about my appearance surface, I pay more attention to my sensations in the present moment, keeping myself calm. When I feel my appearance is "not good enough" in some aspects, I tell myself others feel this too. I know my appearance is imperfect, but I still love myself.

(6) When I encounter difficulties, I strive to keep my emotions as stable as possible and view the challenge with the right perspective; I see my difficulties as part of life that everyone experiences; I believe in myself, grant myself courage and strength, and I face them positively. When others comment on my appearance, I calm my mind and respond objectively with balance; When I realize my appearance is imperfect, I tell myself: most people are imperfect; I maintain self-confidence and accept my physical flaws.

(7) When unpleasant memories surface, I rarely dwell on the past; instead, I focus more on my present feelings and attend to the current experience with an open, accepting, and balanced attitude; I never feel alone, others experience this too; I face it with strength and wait patiently. When others comment on my appearance, I maintain an open, accepting, and balanced attitude; When I realize I have flaws in my appearance, I tell myself everyone has physical imperfections; I consistently treat my physical shortcomings with tolerance.

(8) Whenever I think about my shortcomings, I analyze and accept them calmly rather than criticizing or denying them; I know most people are like me, everyone has flaws; Although I have many shortcomings and imperfections, I try to accept and tolerate them, and I still love myself. When I think about my physical flaws, I attempt to accept my feelings at that moment; When troubled by appearance issues, I feel I am not alone, others experience this too; I strive to be tolerant of my physical flaws and shortcomings.

(9) When criticized or questioned, I try to view the critique comprehensively and objectively, attempting to accept my feelings at that moment; I understand there are no perfect people in this world, most people cannot do everything well; I maintain an attitude of tolerance towards myself. When I think about my physical flaws, I analyze them calmly rather than criticizing or denying them. When I feel I don't look very good, I remind myself that most people are dissatisfied with their own appearance. I try to understand and rationally address the aspects of my appearance I dislike.

(10) When misfortune befalls me, I try to face it with a peaceful mindset, striving to be mindful of the present; I believe suffering and setbacks are part of life, essential lessons in the human experience, a shared aspect of humanity; I try to be strong and view all experiences rationally and objectively. When unpleasant memories about my appearance surface, I pay more attention to my sensations in the present moment, keeping myself calm. When I feel my appearance is "not good enough" in some aspects, I tell myself others feel this too. I know my appearance is imperfect, but I still love myself.

Neutral intervention materials:

(1) In an ecosystem, animals are consumers of organic matter. In the early stages of life development, when only cyanobacteria and bacteria existed on Earth, the ecosystem was a two-loop system consisting of producers and decomposers. With the emergence and development of eukaryotes, particularly animals, the two-loop ecosystem evolved into a three-loop system composed of producers, decomposers, and consumers, forming the diverse and vibrant biological world.

(2) The Big Bang is a cosmological model describing the initial conditions of the universe's birth and its subsequent evolution. It has received the broadest and most precise support from scientific research and observation and is one of the most influential theories in modern cosmology. It posits that the universe underwent a period of evolution from hot to cold, during which the cosmic system continuously expanded, evolving from an initial state of extremely high density and temperature to its present state, analogous to a massive explosion.

(3) Bacteria constitute one of the major groups of organisms, belonging to the domain Bacteria, and are the most numerous classes of all living things. Bacterial shapes are quite diverse, mainly spherical (cocci), rod-shaped (bacilli), and spiral-shaped (spirilla). Bacteria are ubiquitous on Earth, reproduce rapidly, and exist in vast numbers. They are crucial decomposers in ecosystems and play a significant role in natural element cycles. Some bacteria oxidize inorganic matter to derive energy for food production; others contain bacteriochlorophyll and can perform photosynthesis. Bacteria have a substantial impact on human activities.

(4) Evolution, in biology, refers to changes in the inherited characteristics of a population across generations. Natural selection, also known as "survival of the fittest," occurs when an organism's inherited traits confer an advantage or disadvantage in the struggle for survival, leading to differences in survival ability and consequently reproductive success. This results in the preservation or elimination of these traits. Evolutionary theory is one of the most important discoveries and scientific achievements in human knowledge. Darwin's theory of evolution, first proposed, revealed the relationships between biological species at a macro level, uncovered the principles and general pathways of species evolution, and proposed the mechanisms and laws of selective evolution.

(5) The cell is the basic structural and functional unit of living organisms. It is known that all organisms except viruses are composed of cells, although viral life activities must also occur within cells to manifest. Cells possess functions such as movement, nutrition, and reproduction. The study of cells is called cell biology. Prokaryotic and eukaryotic cells are the two fundamental types, reflecting two stages of cellular evolution. The main characteristic of prokaryotic cells is the absence of membrane-bound organelles like mitochondria or plastids; their chromosome is typically a single circular DNA molecule without histone proteins or other associated proteins, and they lack a nuclear membrane.

(6) Physics is the discipline that studies the most general laws of matter in motion and the fundamental structure of matter. As the leading discipline among the natural sciences, physics investigates the most fundamental forms of motion and their laws, ranging from the vast cosmos down to elementary particles. Physics is a natural science focused on the study of matter, energy, space, and time, particularly their individual properties and interrelationships. Physics is knowledge about the laws of nature; more broadly, it explores and analyzes natural phenomena to understand their governing rules.

(7) Buoyancy is the upward force exerted by a fluid on an object immersed in it, acting in the opposite direction to gravity. It refers to the net force (resultant of fluid pressure differences on all surfaces) acting on an object wholly or partially submerged in a fluid (liquid or gas). According to the principle of buoyancy (Archimedes' principle), the buoyant force acting on such an object equals the weight of the fluid displaced by the object's submerged volume. This principle is critically important for calculating the density of objects and consequently for the design and construction of submarines and oceangoing vessels.

(8) In chemical reactions, the ability of a substance to lose electrons is termed its reducibility. The strength of reducibility depends on the substance's propensity to lose electrons in chemical reactions. Reducibility is defined relative to oxidizability, which is the ability of a substance to gain electrons. Substances in a high oxidation state generally possess oxidizability (e.g., some non-metal elements and high-valent metals). Substances in a low oxidation state generally possess reducibility (e.g., some metal elements and non-metal anions). Substances in an intermediate oxidation state often exhibit both reducibility and oxidizability.

(9) The stability of a chemical substance generally refers to the difficulty with which it undergoes changes under realistic conditions. Good stability means the substance is resistant to change. "Realistic conditions" refer to its environment. Most of these changes are chemical (e.g., reaction with oxygen, spontaneous decomposition, reaction with atmospheric moisture), some are physical (e.g., volatilization, precipitation, concentration), and others involve biological factors (e.g., mold growth). Stability can be categorized into thermal stability, photochemical stability, and redox stability. The less reactive a substance is, the better its chemical stability.

(10) Biology is the science that explores life phenomena and the laws governing life activities, serving as a fundamental discipline within the natural sciences. In ancient times, before the development of natural science, people were bewildered by the dazzling variety and brilliance of living things. They often viewed the living and non-living as two entirely distinct and unrelated realms, believing life did not obey the motion laws of non-living matter. Many attributed various life phenomena to the action of a non-material force, the "vital force". These unfounded speculations have gradually been abandoned with the advancement of biology and hold no ground in modern biology.

Table S1 Power, relative bias, and width of credible interval of the fixed effect of the cross-lagged parameters

| *N* | *T* | Power | | Rbias | | Width | |
| --- | --- | --- | --- | --- | --- | --- | --- |
|  |  | b=0.1 | b=0.3 | b=0.1 | b=0.3 | b=0.1 | b=0.3 |
| 50 | 20 | **0.581** | 1.000 | 0.002 | -0.012 | 0.182 | 0.175 |
|  | 50 | **0.780** | 1.000 | -0.010 | -0.024 | 0.141 | 0.137 |
|  | 100 | 0.850 | 1.000 | -0.002 | -0.033 | 0.127 | 0.125 |
|  | 200 | 0.875 | 1.000 | 0.004 | -0.023 | 0.122 | 0.120 |
| 100 | 20 | 0.930 | 1.000 | 0.005 | -0.013 | 0.126 | 0.121 |
|  | 50 | 0.975 | 1.000 | -0.009 | -0.028 | 0.098 | 0.095 |
|  | 100 | 0.990 | 1.000 | 0.004 | -0.026 | 0.088 | 0.087 |
|  | 200 | 0.995 | 1.000 | 0.002 | -0.019 | 0.084 | 0.083 |
| 200 | 20 | 0.995 | 1.000 | 0.001 | -0.020 | 0.089 | 0.085 |
|  | 50 | 1.000 | 1.000 | -0.004 | -0.018 | 0.069 | 0.067 |
|  | 100 | 1.000 | 1.000 | -0.003 | -0.024 | 0.062 | 0.060 |
|  | 200 | 1.000 | 1.000 | -0.001 | -0.017 | 0.059 | 0.058 |

Note. *N* means sample size, *T* means the number of time points. Rbias means relative bias, width means width of credible interval. Reported power, rbias, width values for the two cross-lagged effects were averaged due to their similarity. Results with power lower than 0.8 or rbias outside [-0.1, 0.1] are boldfaced.

Table S2 Power, relative bias, and width of credible interval of the fixed intervention effect on the mean, autoregressive effect, and intra-individual variance based on a random-group design

| *N* | *T* | Power | | | Rbias | | | Width | | |
| --- | --- | --- | --- | --- | --- | --- | --- | --- | --- | --- |
|  |  | mean | AE | IIV | mean | AE | IIV | mean | AE | IIV |
| 30 | 10 | **0.088** | **0.070** | **0.048** | **-0.134** | 0.017 | 0.004 | 0.933 | 0.676 | 0.932 |
|  | 20 | **0.178** | **0.172** | **0.108** | -0.013 | 0.014 | 0.004 | 0.679 | 0.482 | 0.662 |
|  | 40 | **0.358** | **0.302** | **0.164** | -0.035 | 0.003 | -0.019 | 0.498 | 0.364 | 0.492 |
|  | 60 | **0.474** | **0.366** | **0.216** | 0.009 | -0.040 | -0.003 | 0.425 | 0.309 | 0.421 |
|  | 80 | **0.624** | **0.492** | **0.278** | 0.042 | 0.005 | -0.047 | 0.374 | 0.281 | 0.373 |
|  | 100 | **0.704** | **0.550** | **0.344** | 0.044 | -0.016 | 0.020 | 0.345 | 0.261 | 0.345 |
| 60 | 10 | **0.200** | **0.212** | **0.136** | **-0.103** | 0.054 | 0.053 | 0.598 | 0.441 | 0.598 |
|  | 20 | **0.400** | **0.432** | **0.232** | 0.016 | 0.030 | -0.010 | 0.435 | 0.319 | 0.433 |
|  | 40 | **0.668** | **0.648** | **0.376** | -0.047 | 0.007 | -0.013 | 0.324 | 0.241 | 0.323 |
|  | 60 | 0.850 | **0.781** | **0.513** | -0.025 | 0.015 | 0.033 | 0.273 | 0.207 | 0.276 |
|  | 80 | 0.922 | 0.826 | **0.582** | 0.026 | -0.003 | -0.040 | 0.246 | 0.187 | 0.247 |
|  | 100 | 0.966 | 0.890 | **0.700** | 0.005 | -0.003 | 0.008 | 0.226 | 0.174 | 0.227 |
| 100 | 10 | **0.344** | **0.398** | **0.224** | -0.037 | 0.054 | 0.024 | 0.444 | 0.329 | 0.445 |
|  | 20 | **0.660** | **0.664** | **0.382** | 0.005 | 0.029 | 0.001 | 0.325 | 0.240 | 0.323 |
|  | 40 | 0.888 | 0.878 | **0.624** | -0.044 | 0.007 | -0.002 | 0.242 | 0.181 | 0.243 |
|  | 60 | 0.966 | 0.940 | **0.782** | -0.029 | 0.005 | 0.019 | 0.205 | 0.156 | 0.208 |
|  | 80 | 0.996 | 0.975 | 0.832 | 0.002 | 0.002 | -0.019 | 0.184 | 0.141 | 0.186 |
|  | 100 | 1.000 | 0.992 | 0.902 | -0.005 | -0.009 | 0.013 | 0.170 | 0.131 | 0.171 |
| 150 | 10 | **0.560** | **0.584** | **0.344** | -0.057 | 0.029 | 0.033 | 0.355 | 0.264 | 0.356 |
|  | 20 | 0.806 | 0.850 | **0.562** | -0.023 | 0.036 | 0.024 | 0.261 | 0.192 | 0.260 |
|  | 40 | 0.972 | 0.974 | **0.798** | -0.042 | -0.001 | 0.004 | 0.194 | 0.146 | 0.195 |
|  | 60 | 0.998 | 0.988 | 0.926 | 0.007 | -0.021 | 0.026 | 0.165 | 0.126 | 0.167 |
|  | 80 | 1.000 | 0.996 | 0.956 | -0.002 | 0.011 | -0.018 | 0.147 | 0.115 | 0.149 |
|  | 100 | 1.000 | 1.000 | 0.988 | 0.019 | -0.005 | 0.020 | 0.136 | 0.107 | 0.138 |
| 200 | 10 | **0.652** | **0.692** | **0.444** | -0.042 | 0.000 | 0.050 | 0.304 | 0.226 | 0.305 |
|  | 20 | 0.898 | 0.926 | **0.726** | -0.012 | 0.031 | 0.029 | 0.223 | 0.165 | 0.224 |
|  | 40 | 0.996 | 0.992 | 0.936 | -0.032 | 0.002 | 0.017 | 0.166 | 0.125 | 0.168 |
|  | 60 | 0.998 | 1.000 | 0.968 | 0.006 | -0.016 | 0.023 | 0.141 | 0.109 | 0.144 |
|  | 80 | 1.000 | 1.000 | 0.990 | -0.001 | 0.006 | -0.003 | 0.126 | 0.099 | 0.129 |
|  | 100 | 1.000 | 1.000 | 0.998 | 0.010 | -0.003 | 0.022 | 0.117 | 0.092 | 0.119 |
| 300 | 10 | 0.812 | 0.874 | **0.636** | -0.053 | 0.030 | 0.031 | 0.245 | 0.182 | 0.246 |
|  | 20 | 0.974 | 0.994 | 0.884 | -0.031 | 0.011 | 0.008 | 0.180 | 0.134 | 0.180 |
|  | 40 | 1.000 | 1.000 | 0.984 | 0.004 | 0.012 | -0.006 | 0.135 | 0.102 | 0.135 |
|  | 60 | 1.000 | 1.000 | 0.994 | 0.004 | -0.012 | 0.015 | 0.114 | 0.088 | 0.117 |
|  | 80 | 1.000 | 1.000 | 1.000 | -0.001 | -0.004 | -0.001 | 0.102 | 0.080 | 0.104 |
|  | 100 | 1.000 | 1.000 | 1.000 | 0.000 | -0.001 | 0.003 | 0.095 | 0.075 | 0.096 |
| 400 | 10 | 0.926 | 0.942 | **0.778** | -0.049 | 0.040 | 0.038 | 0.211 | 0.156 | 0.212 |
|  | 20 | 0.998 | 0.996 | 0.962 | -0.020 | 0.026 | 0.034 | 0.155 | 0.115 | 0.156 |
|  | 40 | 1.000 | 1.000 | 1.000 | -0.013 | -0.002 | 0.018 | 0.116 | 0.088 | 0.117 |
|  | 60 | 1.000 | 1.000 | 1.000 | 0.000 | -0.008 | 0.013 | 0.099 | 0.076 | 0.101 |
|  | 80 | 1.000 | 1.000 | 1.000 | 0.000 | -0.003 | 0.001 | 0.088 | 0.069 | 0.090 |
|  | 100 | 1.000 | 1.000 | 1.000 | 0.005 | -0.003 | 0.018 | 0.082 | 0.065 | 0.083 |

Note. *N* means sample size (*N*/2 means sample size for each group under a balanced random -group design), *T* means the number of time points before and during EMI, rbias means relative bias, width means width of credible interval, mean means the intervention effect on the mean level, AE means the intervention effect on the autoregressive effect, IIV means the intervention effect on the intra-individual variance. Results with power lower than 0.8 or rbias outside [-0.1, 0.1] are boldfaced.

Table S3 Results of Model 2 in the EMA study

| Parameters | | Unstandardized Estimates | | | Standardized Estimates | | |
| --- | --- | --- | --- | --- | --- | --- | --- |
|  |  | *b* | SD | 95%CI | $\beta$ | SD | 95%CI |
| Fixed effect | ${AASMU}_{i.t-1}^{(w)}$→${AASMU}_{i.t}^{(w)}(\gamma_{10})$ | **0.523** | **0.216** | **[0.097, 0.945]** | **2.103** | **0.884** | **[0.392, 3.830]** |
|  | ${SMU}_{i.t-1}^{(w)}$→${SMU}_{i.t}^{(w)}(\gamma_{40})$ | -0.065 | 0.187 | [-0.426, 0.309] | -0.332 | 0.948 | [-2.157, 1.528] |
|  | ${SMU}_{i.t-1}^{(w)}$→${AASMU}_{i.t}^{(w)}(\gamma_{20})$ | -0.002 | 0.023 | [-0.048, 0.043] | -0.099 | 0.912 | [-1.901, 1.697] |
|  | ${AASMU}_{i.t-1}^{(w)}$→${SMU}_{i.t}^{(w)}(\gamma_{30})$ | -0.004 | 0.054 | [-0.103, 0.102] | -0.117 | 1.876 | [-3.767, 3.568] |
|  | Time→${AASMU}_{i.t}^{(w)}(\gamma_{50})$ | -0.334 | 0.278 | [-0.896, 0.199] | -0.960 | 0.784 | [-0.256, 0.570] |
|  | Time→${SMU}_{i.t}^{(w)}(\gamma_{60})$ | 0.015 | 0.057 | [-0.100, 0.118] | 0.483 | 1.993 | [-3.571, 4.279] |
|  | $\boldsymbol{AASMU}_{\boldsymbol{i}\mathbf{.}\boldsymbol{t}}^{\mathbf{(}\boldsymbol{w}\mathbf{)}}$**↔**$\boldsymbol{SMU}_{\boldsymbol{i}\mathbf{.}\boldsymbol{t}}^{\left( \boldsymbol{w} \right)}\mathbf{(}\boldsymbol{\gamma}_{\mathbf{90}}\mathbf{)}$ | **0.457** | **0.172** | **[****0.116, 0.788]** | **4.725** | **2.032** | **[1.229, 9.283]** |
|  | **residual variance of AASMU (**$\boldsymbol{\gamma}_{\boldsymbol{70}}$**)** | **5.129** | **0.683** | **[3.771, 6.457]** | **4.909** | **0.679** | **[3.546, 6.195]** |
|  | residual variance of SMU ($\gamma_{80}$) | 2.347 | 2.602 | [-2.818, 7.367] | 0.554 | 0.612 | [-0.651, 1.749] |
|  | **SC→**$\boldsymbol{\mu}_{\boldsymbol{AASMU}\mathbf{.}\boldsymbol{i}}$**(**$\boldsymbol{\gamma}_{\mathbf{010}}$**)** | **8.426** | **2.075** | **[****4.461, 12.550]** | **0.211** | **0.051** | **[0.110, 0.310]** |
|  | HL→$\mu_{AASMU.i}$($\gamma_{011}$) | -2.353 | 3.801 | [-9.774, 4.977] | -0.042 | 0.067 | [-0.172, 0.087] |
|  | **SEC→**$\boldsymbol{\mu}_{\boldsymbol{AASMU}\mathbf{.}\boldsymbol{i}}$**(**$\boldsymbol{\gamma}_{\mathbf{012}}$**)** | **-5.697** | **2.669** | **[****-10.860, -0.403]** | **-0.144** | **0.067** | **[-0.268, -0.010]** |
|  | HL→$\mu_{SMU.i}$($\gamma_{021}$) | -25.579 | 18.116 | [-61.169, 10.050] | -0.071 | 0.050 | [-0.169, 0.028] |
|  | HL→$\phi_{11.i}$ ($\gamma_{11}$) | -0.062 | 0.059 | [-0.177, 0.054] | -0.073 | 0.070 | [-0.208, 0.063] |
|  | HL→$\phi_{22.i}$ ($\gamma_{41}$) | 0.075 | 0.051 | [-0.027, 0.175] | 0.112 | 0.078 | [-0.038, 0.262] |
|  | HL→$\phi_{12.i}$ ($\gamma_{21}$) | 0.001 | 0.006 | [-0.012, 0.013] | 0.010 | 0.073 | [-0.136, 0.154] |
|  | HL→$\phi_{21.i}$ ($\gamma_{31}$) | 0.001 | 0.016 | [-0.030, 0.030] | 0.005 | 0.160 | [-0.309, 0.319] |
|  | HL→$\phi_{13.i}$ ($\gamma_{51}$) | 0.040 | 0.076 | [-0.108, 0.193] | 0.033 | 0.063 | [-0.090, 0.158] |
|  | HL→$\phi_{23.i}$ ($\gamma_{61}$) | -0.005 | 0.017 | [-0.035, 0.029] | -0.046 | 0.171 | [-0.374, 0.307] |
|  | **HL→**$\log\left( \boldsymbol{\pi}_{\mathbf{1.}\boldsymbol{i}} \right)$**(**$\boldsymbol{\gamma}_{\mathbf{71}}$**)** | **-0.418** | **0.187** | **[****-0.782, -0.050]** | **-0.118** | **0.052** | **[-0.216, -0.014]** |
|  | HL→$\log\left( \pi_{2.i} \right)$($\gamma_{81}$) | 1.310 | 0.712 | [-0.070, 2.714] | 0.091 | 0.049 | [-0.005, 0.186] |
|  | **HL→**$\boldsymbol{cov}_{\mathbf{1.}\boldsymbol{i}}$**(**$\boldsymbol{\gamma}_{\mathbf{91}}$**)** | **-0.137** | **0.047** | **[****-0.229, -0.044]** | **-0.415** | **0.166** | **[-0.790, -0.137]** |
| Residual variance | **AASMU (**$\boldsymbol{u}_{\mathbf{01}\boldsymbol{i}}$**)** | **253.713** | **28.395** | **[204.306,316.691]** | **0.924** | **0.025** | **[0.868, 0.964]** |
|  | **SMU (**$\boldsymbol{u}_{\mathbf{02}\boldsymbol{i}}$**)** | **11045.667** | **1238.432** | **[8899.122,13778.480]** | **0.995** | **0.008** | **[0.971, 1.000]** |
|  | $\boldsymbol{AASMU}_{\boldsymbol{i}\mathbf{.}\boldsymbol{t}\mathbf{-1}}^{\mathbf{(}\boldsymbol{w}\mathbf{)}}$**→**$\boldsymbol{AASMU}_{\boldsymbol{i}\mathbf{.}\boldsymbol{t}}^{\mathbf{(}\boldsymbol{w}\mathbf{)}}\mathbf{(}\boldsymbol{u}_{\mathbf{11}\boldsymbol{i}}\mathbf{)}$ | **0.062** | **0.011** | **[0.042, 0.087]** | **0.994** | **0.012** | **[0.957, 1.000]** |
|  | $\boldsymbol{SMU}_{\boldsymbol{i}\mathbf{.}\boldsymbol{t}\mathbf{-1}}^{\mathbf{(}\boldsymbol{w}\mathbf{)}}$**→**$\boldsymbol{SMU}_{\boldsymbol{i}\mathbf{.}\boldsymbol{t}}^{\mathbf{(}\boldsymbol{w}\mathbf{)}}\mathbf{(}\boldsymbol{u}_{\mathbf{22}\boldsymbol{i}}\mathbf{)}$ | **0.038** | **0.009** | **[0.024, 0.059]** | **0.987** | **0.019** | **[0.931, 1.000]** |
|  | $\boldsymbol{SMU}_{\boldsymbol{i}\mathbf{.}\boldsymbol{t}\mathbf{-1}}^{\mathbf{(}\boldsymbol{w}\mathbf{)}}$**→**$\boldsymbol{AASMU}_{\boldsymbol{i}\mathbf{.}\boldsymbol{t}}^{\mathbf{(}\boldsymbol{w}\mathbf{)}}\mathbf{(}\boldsymbol{u}_{\mathbf{12}\boldsymbol{i}}\mathbf{)}$ | **0.001** | **0.000** | **[0.000, 0.001]** | **0.998** | **0.008** | **[0.972, 1.000]** |
|  | $\boldsymbol{AASMU}_{\boldsymbol{i}\mathbf{.}\boldsymbol{t}\mathbf{-1}}^{\mathbf{(}\boldsymbol{w}\mathbf{)}}$**→**$\boldsymbol{SMU}_{\boldsymbol{i}\mathbf{.}\boldsymbol{t}}^{\mathbf{(}\boldsymbol{w}\mathbf{)}}\mathbf{(}\boldsymbol{u}_{\mathbf{21}\boldsymbol{i}}\mathbf{)}$ | **0.001** | **0.000** | **[0.000, 0.001]** | **0.988** | **0.034** | **[0.874, 1.000]** |
|  | **Time→**$\boldsymbol{AASMU}_{\boldsymbol{i}\mathbf{.}\boldsymbol{t}}^{\mathbf{(}\boldsymbol{w}\mathbf{)}}\mathbf{(}\boldsymbol{u}_{\mathbf{13}\boldsymbol{i}}\mathbf{)}$ | **0.124** | **0.026** | **[0.080, 0.181]** | **0.998** | **0.007** | **[0.975, 1.000]** |
|  | **Time→**$\boldsymbol{SMU}_{\boldsymbol{i}\mathbf{.}\boldsymbol{t}}^{\mathbf{(}\boldsymbol{w}\mathbf{)}}\mathbf{(}\boldsymbol{u}_{\mathbf{23}\boldsymbol{i}}\mathbf{)}$ | **0.001** | **0.000** | **[0.000, 0.001]** | **0.986** | **0.043** | **[0.846, 1.000]** |
|  | $\boldsymbol{AASMU}_{\boldsymbol{i}\mathbf{.}\boldsymbol{t}}^{\mathbf{(}\boldsymbol{w}\mathbf{)}}$**↔**$\boldsymbol{SMU}_{\boldsymbol{i}\mathbf{.}\boldsymbol{t}}^{\left( \boldsymbol{w} \right)}\mathbf{(}\boldsymbol{u}_{\boldsymbol{cov}\mathbf{.}\boldsymbol{i}}\mathbf{)}$ | **0.008** | **0.004** | **[****0.001, 0.018]** | **0.827** | **0.157** | **[0.377, 0.981]** |
|  | **residual variance of AASMU (**$\boldsymbol{u}_{\boldsymbol{\pi}\boldsymbol{1.i}}$**)** | **1.077** | **0.130** | **[0.856, 1.368]** | **0.986** | **0.013** | **[0.953, 1.000]** |
|  | **residual variance of SMU (**$\boldsymbol{u}_{\boldsymbol{\pi}\boldsymbol{2.i}}$**)** | **17.875** | **1.787** | **[14.719, 21.714]** | **0.992** | **0.009** | **[0.965, 1.000]** |

Note. Unstandardized estimations were recommended because many cases were removed by Mplus when calculating standardized estimations. SD = standard deviation of posterior distribution, 95 % CI = 95 % credible interval. The significant estimates were highlighted with boldface.

**
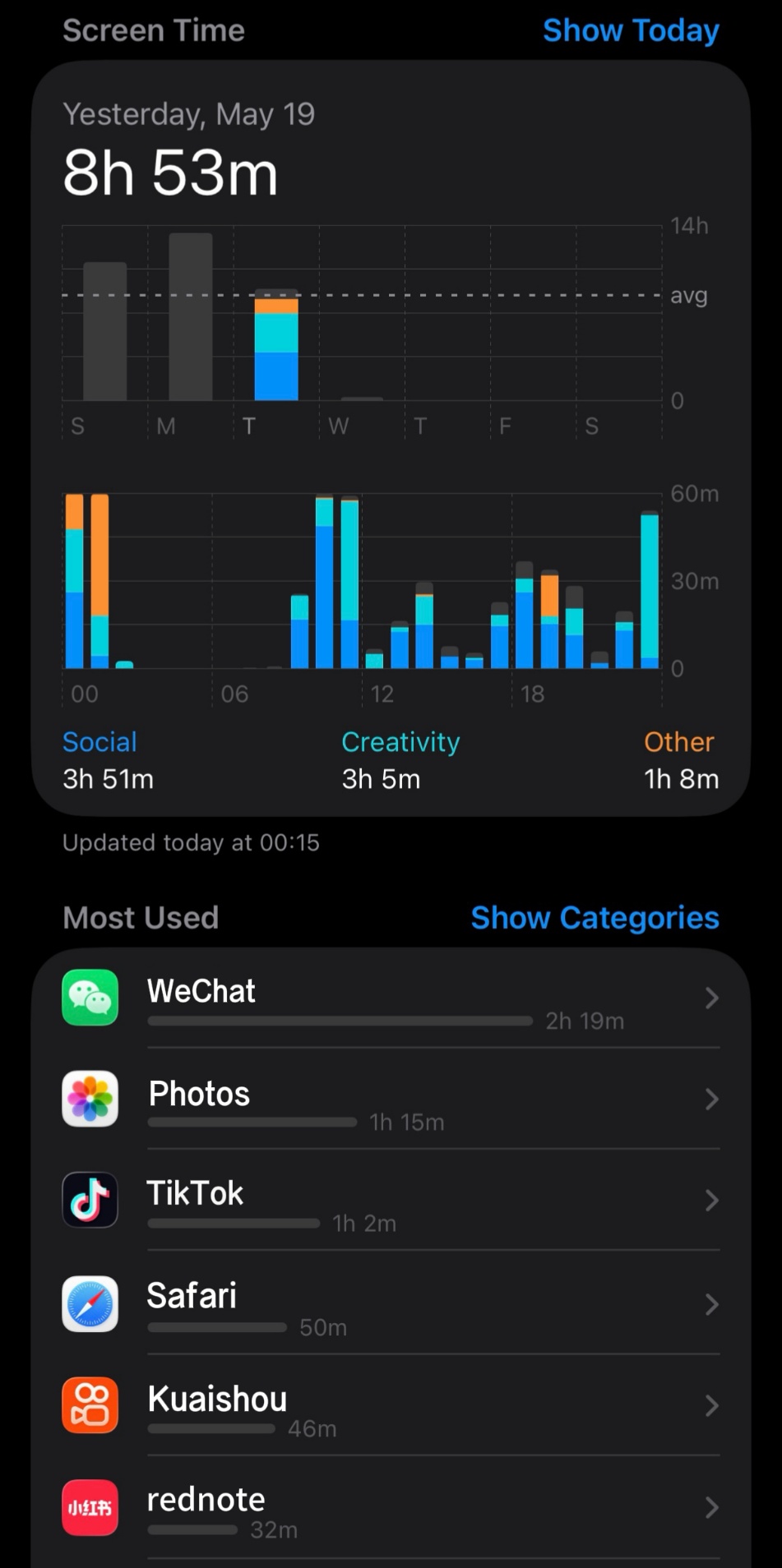
**

Figure S1 Example screenshot of daily screen usage time


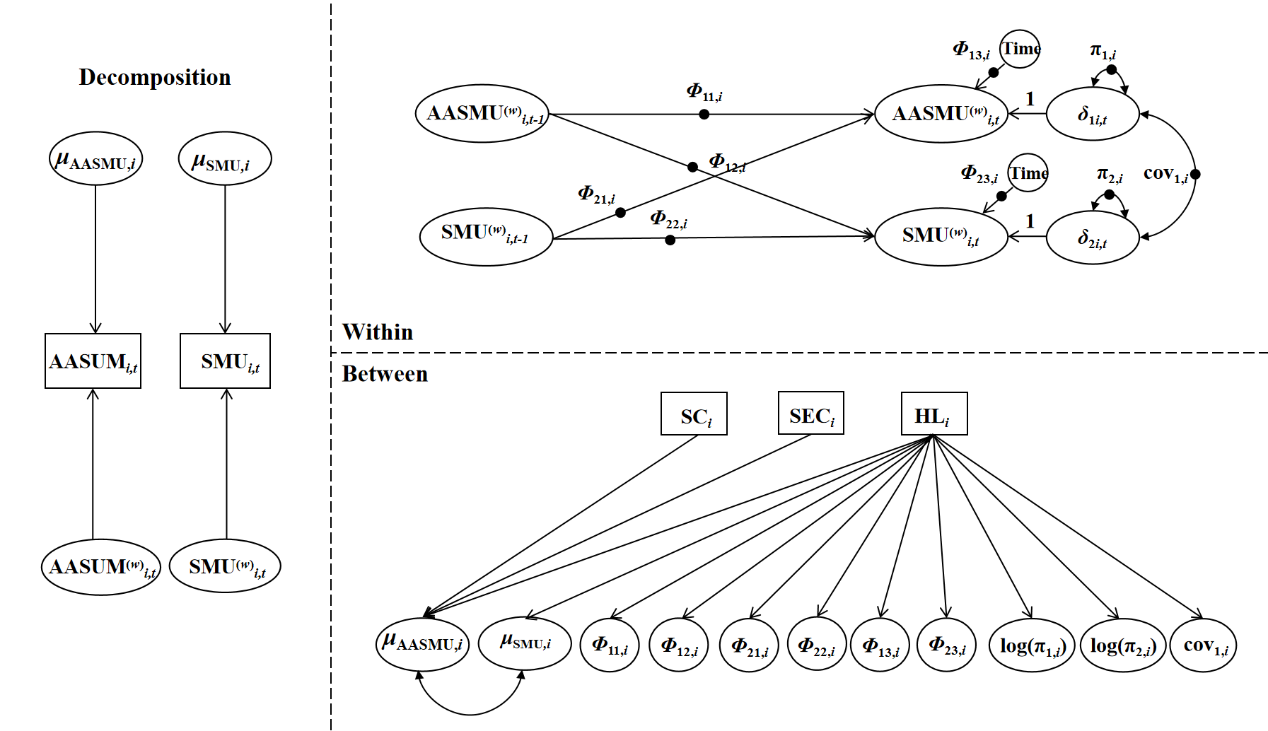


Figure S2 Analytical model of the dynamic relationship between the different levels of each variable in Model 2.

Latent variables are represented by circles, while observed variables are represented by squares. (*w*) denotes the estimate at the time-point level (within-person level), with the subscript *i* denotes the subject, *t* denotes the time point and *μ* denotes the mean. AASMU and SMU represent the variables appearance anxiety for social media users and social media use respectively, while SC, HL, and SEC represent: social comparison tendency, healthy lifestyle, and level of self-compassion, respectively. Solid black dots on each path indicate that the corresponding coefficients are random.
